# Trends and projections of inflammatory bowel disease at the global, regional and national levels, 1990–2050: a bayesian age-period-cohort modeling study

**DOI:** 10.1186/s12889-023-17431-8

**Published:** 2023-12-14

**Authors:** Jia-Li Zhou, Jia-Chen Bao, Xu-Ying Liao, Yi-Jia Chen, Lin-Wei Wang, Yan-Yun Fan, Qin-Yu Xu, Lan-Xiang Hao, Kun-Jian Li, Ming-Xian Liang, Tian-Hui Hu, Zheng-Jin Liu, Yi-Qun Hu

**Affiliations:** 1 School of Medicine, Xiamen University, Xiamen, 361102 Fujian Province China; 2 The Third Clinical Medical College, Fujian Medical University, Fuzhou, 350004 Fujian China; 3 Department of Gastroenterology, Zhongshan Hospital of Xiamen University, School of Medicine, Xiamen University, 201 Hubin South Road, Xiamen, Fujian Province People’s Republic of China 361004; 4 Department of Ultrasound, Zhongshan Hospital of Xiamen University, School of Medicine, Xiamen University, 201 Hubin South Road, Xiamen, Fujian Province People’s Republic of China 361004; 5 Department of Clinical Laboratory, Zhongshan Hospital of Xiamen University, School of Medicine, Xiamen University, 201 Hubin South Road, Xiamen, Fujian Province People’s Republic of China 361004; 6 Anti Cancer Research Center of Xiamen University School of Medicine, Zhongshan Hospital of Xiamen University, 201 Hubin South Road, Xiamen, Fujian Province People’s Republic of China 361004

**Keywords:** Global burden of disease study database, Socio-demographic index, Socioeconomics, Epidemiological stages, Inflammatory bowel diseases

## Abstract

**Background:**

Inflammatory bowel disease (IBD) is a global health concern with varying levels and trends across countries and regions. Understanding these differences is crucial for effective prevention and treatment strategies.

**Methods:**

Using data from the 2019 Global Burden of Disease study, we examine IBD incidence, mortality, and disability-adjusted life years (DALYs) rates in 198 countries from 1990 to 2019. To assess changes in the burden of IBD, estimated annual percentage changes (EAPC) were calculated, and a Bayesian age-period-cohort model was used to predict the future 30-year trends of IBD.

**Results:**

In 2019, there were 405,000 new IBD cases globally (95% uncertainty interval (UI) 361,000 to 457,000), with 41,000 deaths (95% UI 35,000 to 45,000) and 1.62million DALYs (95% UI 1.36–1.92million). The global age-standardized incidence rate in 2019 was 4.97 per 100,000 person-years (95% UI 4.43 to 5.59), with a mortality rate of 0.54 (95% UI 0.46 to 0.59) and DALYs rate of 20.15 (95% UI 16.86 to 23.71). From 1990 to 2019, EAPC values for incidence, mortality, and DALYs rates were − 0.60 (95% UI − 0.73 to − 0.48), − 0.69 (95% UI − 0.81 to − 0.57), and − 1.04 (95% UI − 1.06 to − 1.01), respectively. Overall, the burden of IBD has shown a slow decline in recent years. In SDI stratification, regions with higher initial SDI (high-income North America and Central Europe) witnessed decreasing incidence and mortality rates with increasing SDI, while regions with lower initial SDI (South Asia, Oceania, and Latin America) experienced a rapid rise in incidence but a decrease in mortality with increasing SDI. Predictions using a Bayesian model showed lower new cases and deaths from 2020 to 2050 than reference values, while the slope of the predicted incidence-time curve closely paralleled that of the 2019 data.

**Conclusion:**

Increasing cases, deaths, and DALYs highlight the sustained burden of IBD on public health. Developed countries have stabilized or declining incidence rates but face high prevalence and societal burden. Emerging and developing countries experience rising incidence. Understanding these changes aids policymakers in effectively addressing IBD challenges in different regions and economic contexts.

**Supplementary Information:**

The online version contains supplementary material available at 10.1186/s12889-023-17431-8.

## Introduction

Inflammatory Bowel Diseases(IBD) are non-infectious chronic gastrointestinal inflammations, including Crohn’s Disease (affecting any part of the gastrointestinal tract from the mouth to the anus), Ulcerative Colitis (limited to the colon mucosa), and Unclassified Colitis. IBD is characterized by a relapsing-remitting course and heterogeneous clinical manifestations [1]. This chronic condition cannot be cured and typically requires lifelong care and medication.

In the 1950s, Western countries experienced a peak in IBD incidence, while there were few reported cases in Asia, Africa, and Latin America [2]. Since the twenty-first century, Western countries have seen a stabilization of incidence rates, whereas emerging industrialized countries in Asia, South America, and the Middle East have seen a continuous increase. This variation may be attributed to different stages of IBD across regions and countries at different times [3]. However, IBD is also associated with complex complications, an unpredictable relapsing-remitting course, and expensive treatment costs [4, 5]. The increasing incidence will significantly contribute to the global burden of the disease. Therefore, a comparable and systematic analysis of IBD incidence, mortality, and DALYs rates across different countries and regions can assist in the allocation of healthcare resources at a macro level.

In recent years, several studies have reported the global burden of IBD from 1990 to 2017. Here, we provide additional insights into the burden of IBD in 198 countries and regions from 1990 to 2019, using the latest estimates from the Global Burden of Disease (GBD) study in 2019. We examine the incidence, mortality, and DALYs rates. We also analyze the impact of Socio-Demographic Index (SDI) on the burden of IBD across regions and countries. Furthermore, we predict the future disease burden of IBD using a Bayesian age-period-cohort model.

## Methods

### Data

Global burden data on IBD can be obtained from the GBD database [6], which comprehensively estimates the burden of 369 diseases and injuries, as well as 87 attributable risk factors, by gender, age, region, and country. Based on the SDI, 204 countries and regions are categorized into five regions: low, low-middle, middle, middle-high, and high [7]. Considering geographical consistency, these countries and regions can be further grouped into 17 regions, such as Western Pacific and Central Europe. The data results are presented as numbers with 95% uncertainty intervals (UI).

### Statistical analysis

Incidence rates, mortality rates, DALYs [4], and their corresponding age-standardized rates (ASR) are used to assess the trends of IBD. DALYs, which are calculated by summing years lived with disability (YLDs) and years of life lost (YLLs), provide a comprehensive measure of disease burden [4].When comparing incidence rates and mortality rates, ASR is a rate of a disease incidence or mortality when adjusted for age distribution within a population. This adjustment allows for more accurate comparisons of rates over time or between different populations:

$$\textrm{ASR}=\sum \textrm{ASR}=\frac{\sum_{i=1}^A aiwi}{\sum_{i=1}^A wi}\times \textrm{100,000}$$

*a*i represents the specific age ratio for the i age group, wi represents the number or weight of the corresponding age group in the selected reference standard population, and A represents the quantity of age groups [8]. We reported rates per 100,000 population for each ratio and provided corresponding 95% UI. Additionally, we used EAPC to quantify the time trends of age-standardized incidence rates, mortality rates, and DALY rates for IBD from 1990 to 2019. EAPC was calculated using a regression model based on the natural logarithm of the rates:

$$\textrm{y}=\upalpha +\upbeta \textrm{x}+\upvarepsilon$$

The EAPC is calculated as 100 × (exp(β) − 1), x is calendar years, y is ln(ASR). It can be obtained from the linear regression model, and its 95% confidence interval (CI) can also be derived [9]. If both the predicted value of EAPC and the lower bound of its 95% CI are greater than 0, it is considered an increasing trend in ASR. Conversely, if the predicted value of EAPC and the upper bound of its 95% CI are both less than 0, it indicates a decreasing trend in ASR. Otherwise, the ASR is considered to remain stable over time [9].

### Model

We employed a bayesian age-period-cohort [10] (BAPC) model with nested Laplace approximation to predict the future burden pattern of IBD. This model has been shown to have better accuracy compared to other forecasting models in previous studies [10–12]. The BAPC model is based on the age-period-cohort [13] (APC) model, which assumes an association between the incidence or mortality rates and age structure and population size. In essence, the APC model can be understood as a logarithmic linear Poisson model.

$$\textrm{nij}=\log \left(\uplambda \textrm{i}\textrm{j}\right)=\upmu +\upalpha \textrm{i}+\upbeta \textrm{j}+\upgamma \textrm{k}+\upvarepsilon$$

In this model, μ represents the intercept, C represents the random error, α, β and γ represent the coefficients for age, period, and cohort respectively. The coefficients i, j and k represent the effects of age, period, and cohort, respectively. Assuming that the effects of age, period, and cohort are similar for neighboring time points, the BAPC model applies a second-order random walk with inverse gamma prior distributions to the age, period, and cohort effects. To approximate the marginal posterior distribution, the BAPC model uses an integrated nested Laplace approximation. Additionally, to present the forecasting results more effectively, we set the baseline reference, negative reference, and positive reference. The baseline reference is based on the incidence and mortality rates in 2019. The negative reference assumes a 1% annual increase in the incidence and mortality rates from 2019. The positive reference assumes a 1% annual decrease in the incidence and mortality rates from 2019. We calculated the absolute numbers of new cases and deaths based on the changes in mortality rates. The BAPC model was implemented using the BAPC package in R and nested Laplace approximation based on INLA in R (version 4.4.1).

## Results

### Gender and age

#### Gender

**Fig. 1:**
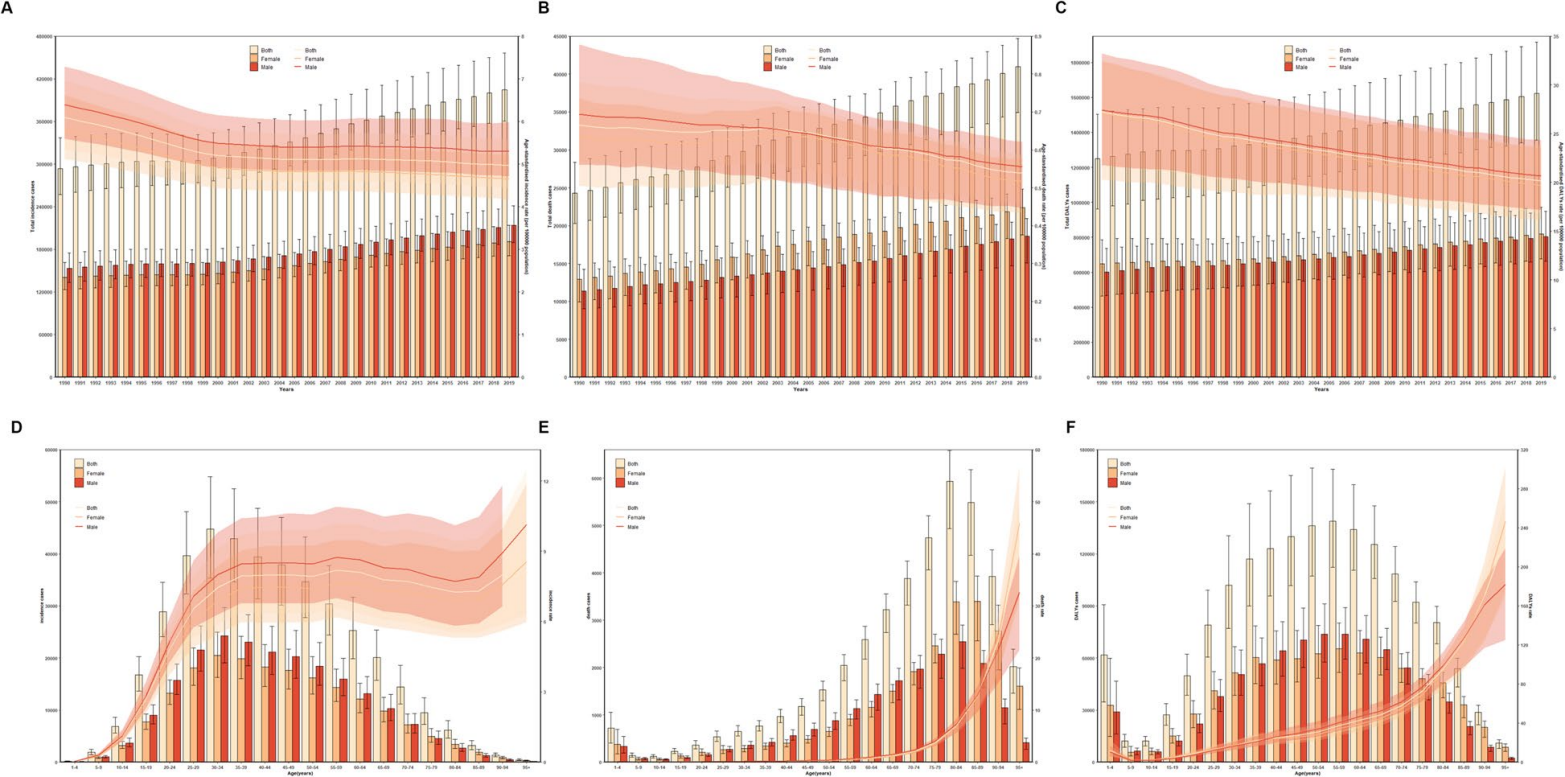
The distribution of inflammatory bowel disease cases globally by age and gender. Global counts and age-standardized incidence (**A**), death (**B**) and DALY (**C**) rates of Inflammatory bowel disease per 100,000 population by sex, from 1990 to 2019. Global counts and age-standardized incidence (**D**), death (**E**) and DALY (**F**) rates of Inflammatory bowel disease per 100,000 population by age and sex, 2019. Error bars indicate the 95% uncertainty intervals (95% UI) for incidence (**A**), death (**B**) and DALYs (**C**). Shading indicates the upper and lower limits of the 95% UI. DALY, disability adjusted life years

In 2019, the estimated number of incident cases for males was 214,000 (95% UI 190,000 to 241,000) and for females was 191,000 (95% UI 171,000 to 214,000) (Fig. 1A). The estimated number of deaths for males was 19,000 (95% UI 15,000 to 21,000) and for females was 22,000 (95% UI 19,000 to 25,000) (Fig. 1B). The estimated DALYs for males were 803,000(95% UI 664,000 to 949,000) and for females were 820,000 (95% UI 677,000 to 972,000) (Fig. 1C).

In 2019, the age-standardized incidence rate per 100,000 person-years was 5.30 (95% CI 4.72 to 5.99) for males and 4.64 (95% CI 4.16 to 5.20) for females (Fig. 1A). The age-standardized mortality rate per 100,000 person-years was 0.56 (95% CI 0.45 to 0.62) for males and 0.52 (95% CI 0.43 to 0.58) for females (Fig. 1B). The age-standardized DALYs rate per 100,000 person-years was 20.64 (95% CI 17.16 to 24.31) for males and 19.70 (95% CI 16.16 to 23.49) for females (Fig. 1C).

From 1990 to 2019, the EAPC for age-standardized incidence rate was − 0.55 (95% CI − 0.68 to − 0.43) for males and − 0.65 (95% CI − 0.78 to − 0.53) for females. The EAPC for age-standardized mortality rate was − 0.78 (95% CI − 0.83 to − 0.72) for males and − 0.64 (95% CI − 0.80 to − 0.47) for females. The EAPC for age-standardized DALYs rate was − 0.99 (95% CI − 1.01 to − 0.97) for males and − 1.09 (95% CI − 1.12 to − 1.06) for females (Table 1).

**Table 1:** The incidence, deaths and DALYs for Inflammatory bowel disease in 2019 for both sexes, EAPC and ASRs by Global Burden from 1990 to 2019

Variables	1990		2019		1990–2019
Incidence (95%Uncertainty Interval)	Counts	ASR per 100,000 population (95%UI)	Counts	ASR per 100,000 population (95%UI)	EAPC in ASR (95% UI)
Overall	293,571.89 (257,425.21 to 336,650.92	6.10 (5.35 to 6.96)	404,552.37 (360,521.17 to 456,478.46)	4.97 (4.43 to 5.59)	−0.60 (− 0.73 to − 0.48)
Males	153,073.97 (133,344.30 to 174,639.60)	6.39 (5.60 to 7.29)	213,814.32 (189,759.04 to 241,303.92)	5.30 (4.72 to 5.99)	−0.55 (− 0.68 to − 0.43)
Females	140,497.92 (123,360.74 to 161,173.20)	5.80 (5.12 to 6.61)	190,738.05 (171,154.23 to 213,778.43)	4.64 (4.16 to 5.20)	−0.65 (− 0.78 to − 0.53)
Death (95%Uncertainty Interval)					
Overall	24,294.72 (20,257.71 to 28,375.62)	0.67 (0.57 to 0.78)	40,998.31 (34,932.99 to 44,660.80)	0.54 (0.46 to 0.59)	−0.69 (−0.81 to − 0.57)
Males	113,47.40 (9002.36 to 14,217.56)	0.69 (0.56 to 0.88)	18,633.15 (15,059.90 to 20,938.34)	0.56 (0.45 to 0.62)	−0.78 (− 0.83 to − 0.72)
Females	12,947.31 (9952.95 to 14,887.69)	0.63 (0.50 to 0.74)	22,365.16 (18,758.61 to 24,838.06)	0.52 (0.43 to 0.58)	−0.64 (− 0.80 to − 0.47)
DALYs (95%Uncertainty Interval)					
Overall	1,248,274.62 (963,014.37 to 1,503,173.72)	27.20 (21.70,32.39)	1,622,498.43 (1,356,295.86 to 1,915,042.45)	20.15 (16.86,23.71))	−1.04 (−1.06 to − 1.01)
Males	600,963.48 (466,033.47 to 736,983.27)	27.40 (21.78,33.22)	802,747.68 (663,562.52 to 949,341.89)	20.64 (17.16,24.31)	−0.99 (−1.01 to − 0.97)
Females	647,311.14 (465,166.24 to 785,220.92)	27.09 (20.30,32.42)	819,750.75 (676,699.64 to 971,584.22)	19.70 (16.16,23.49)	−1.09 (− 1.12 to − 1.06)

From 1990 to 2019, there was an overall declining trend in age-standardized incidence rate in both males and females. We observed a shift around the year 2000, where the decline in age-standardized incidence rate was steeper from 1990 to 2000, followed by a slower decline from 2000 to 2019 (Fig. 1A). Throughout 1990 to 2019, age-standardized incidence rate, mortality rate, and DALYs rate were higher in males compared to females.

From 1990 to 2019, age-standardized mortality rate consistently decreased in males, while in females, it had a slow decline from 1990 to 1998 and even a slight increase from 1998 to 2003 (Fig. 1B). After 2003, both males and females experienced a rapid decline in age-standardized mortality rate. Age-standardized DALYs rate showed a steady decline in both males and females from 1990 to 2019.

#### Age

In 2019, the incidence rate showed a rapid increase from the age group of 1–4 years to 35–39 years. From 35 to 39 years to 80–84 years, the incidence rate entered a fluctuating period, ranging between 6 and 9%. However, in the age group of 85–89 years and beyond, the incidence rate abruptly increased and exceeded 9% (Fig. 1D). The incidence rate among males was consistently higher than females across all age groups. The number of new cases in males was higher than females from 5 to 9 years to 70–74 years. However, from 75 to 79 years to 95 years and beyond, the number of new cases in females surpassed males. Moreover, from 75 to 79 years to 95 years and beyond, the number of deaths (Fig. 1E) and DALYs (Fig. 1F) in females exceeded males.

In 2019, as the age group increased, the mortality rate gradually increased. Before the age group of 85–89 years, the mortality rate among males was slightly higher than females. However, after the age group of 85–89 years, the mortality rate among females was higher than males (Fig. 1E).

In 2019, except for the age groups of 1–4 years to 5–9 years, the DALYs rate showed a declining trend. For the remaining age groups, the DALYs rate increased with age. Females had a higher DALYs rate than males from 1 to 4 years to 40–44 years and from 85 to 89 years to 95 years and beyond. On the other hand, males had a higher DALYs rate than females from 40 to 44 years to 85–89 years.

### Area

#### Global

**Fig. 2:**
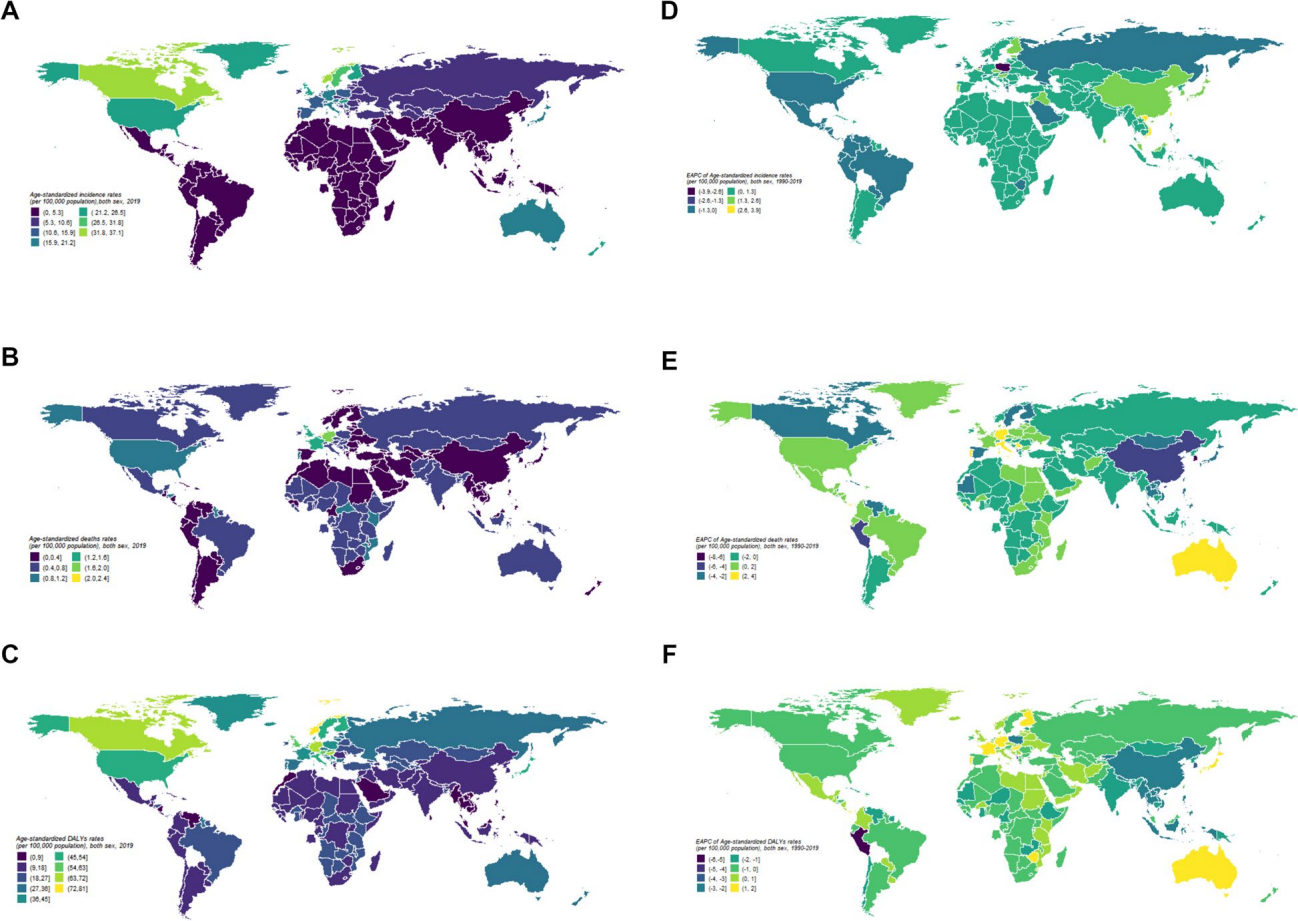
Global age standardized inidence, death, DALYs rate of inflammatory bowel disease. Age-standardized incidence (**A**), death (**B**) and DALY (**C**) rates of Inflammatory bowel disease per 100,000 population both sex, 2019. EACP of age-standardized incidence (**D**), prevalence (**E**) and DALY(F) rates of Inflammatory bowel disease per 100,000 population both sex, 1990–2019. Maps were generated using R software (version 4.0.3) and ggplot2 package

At a global level, in 2019, there were 405,000 (95% UI 361,000 to 457,000) new cases, 41,000 (95% UI 35,000 to 45,000) deaths, and 1.62million (95% UI 1.36million to 1.92million) DALYs (Table 1). The global age-standardized incidence rate in 2019 was 4.97 (95% UI 4.43 to 5.59) per 100,000 person-years (Fig. 2A). The global age-standardized mortality rate in 2019 was 0.54 (95% UI 0.46 to 0.59) per 100,000 person-years (Fig. 2B). The global age-standardized DALYs rate in 2019 was 20.15 (95% UI 16.86 to 23.71) per 100,000 person-years (Fig. 2C).

From 1990 to 2019, the global age-standardized incidence rate had an EAPC of − 0.60 (95% UI − 0.73 to − 0.48) (Fig. 2D). The global age-standardized mortality rate had an EAPC of − 0.69 (95% UI − 0.81 to − 0.57) from 1990 to 2019 (Fig. 2E). The global age-standardized DALYs rate had an EAPC of − 1.04 (95% UI − 1.06 to − 1.01) from 1990 to 2019 (Fig. 2F).

#### Region

**Fig. 3:**
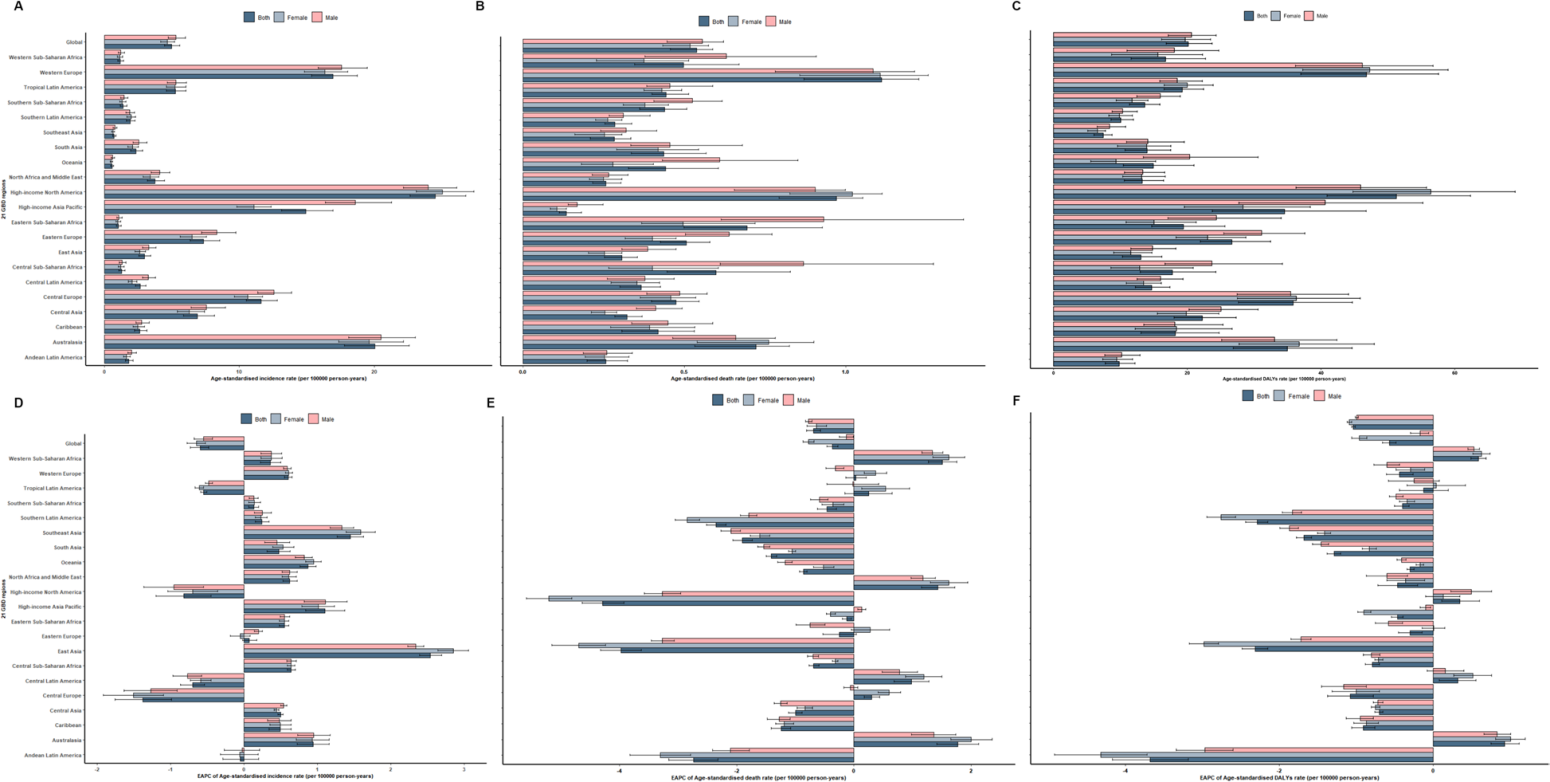
Inflammatory bowel disease by sex in 21 global burden-of-disease regions. Age-standardized incidence (**A**), death (**B**) and DALY (**C**) rates of Inflammatory bowel disease per 100,000 population for 21 Global Burden Disease regions by sex, 2019. EAPC of Age-standardized incidence (**D**), death (**E**) and DALY (**F**) rates of Inflammatory bowel disease per 100,000 population for 21 Global Burden Disease regions by sex, 1990–2019.Error bars indicate the 95% uncertainty intervals (95% UI) for incidence (**A**), death (**B**) and DALYs (**C**). DALY disability adjusted life years

In 2019, the highest age-standardized incidence rate occurred in high-income North America at 24.51 (95% UI 22.65 to 26.77) per 100,000 person-years, followed by Australasia at 20.03 (95% UI 17.79 to 22.57) and Western Europe at 16.94 (95% UI 15.33 to 18.73) (Fig. 3A). The lowest age-standardized incidence rate occurred in Oceania at 0.56 (95% UI 0.46 to 0.67), Southeast Asia at 0.70 (95% UI 0.59 to 0.84), and Eastern Sub-Saharan Africa at 1.04 (95% UI 0.88 to 1.25) (Fig. 3A).

In 2019, the highest age-standardized mortality rate occurred in Eastern Europe at 0.64 (95% UI 0.50 to 0.77) per 100,000 person-years, followed by South Asia at 0.44 (95% UI 0.33 to 0.57) and the Caribbean at 0.42 (95% UI 0.30 to 0.53) (Fig. 3B). The lowest age-standardized mortality rate occurred in Central Europe at 0.47 (95% UI 0.40 to 0.54), North Africa and the Middle East at 0.26 (95% UI 0.22 to 0.30), and Tropical Latin America at 0.44 (95% UI 0.40 to 0.51) (Fig. 3B).

In 2019, the highest age-standardized DALYs rate occurred in high-income North America at 51.27% (95% UI 40.90 to 62.35%) per 100,000 person-years, followed by Western Europe at 46.79% (95% UI 36.98 to 57.56%) and Central Europe at 35.84% (95% UI 27.66 to 44.67%) (Fig. 3C). The lowest age-standardized DALYs rate occurred in Southeast Asia at 7.45% (95% UI 6.06 to 8.76%), Tropical Latin America at 9.83% (95% UI 7.75 to 12.19%), and Southern Latin America at 10.06% (95% UI 8.61 to 11.94%) (Fig. 3C).

In 2019, for most regions, the age-standardized incidence rate (except for high-income North America and Southern Latin America) (Fig. 3A) and the age-standardized mortality rate (except for Australasia and Western Europe) (Fig. 3B) were higher in males than in females. Except for high-income North America, Western Europe, Australasia, Central Europe, Tropical Latin America, and the Caribbean region, the age-standardized DALYs rate in males was higher than in females (Fig. 3C).

From 1990 to 2019, the age-standardized incidence rate increased in most regions, with the highest EAPC observed in East Asia at 2.54% (95% UI 2.39 to 2.69%) (Fig. 3E). From 1990 to 2019, the age-standardized mortality rate and age-standardized DALYs rate decreased in most regions, with the largest EAPC decrease in the age-standardized mortality rate observed in high-income Asia-Pacific at − 4.29% (95% UI − 4.64% to − 3.93%) (Fig. 3D), and the largest EAPC decrease in the age-standardized DALYs rate observed in Andean Latin America at − 3.68% (95% UI − 4.17% to − 3.18%) (Fig. 3F).

#### Country

In 2019, the United States had the highest number of new cases (85,000, 95% UI 78,000 to 95,000), followed by China (52,000, 95% UI 44,000 to 61,000) and India (32,000, 95% UI 26,000 to 39,000). The United States also had the highest number of deaths (6000, 95% UI 5000 to 7000), followed by China (5000, 95% UI 4000 to 5000) and Germany (4000, 95% UI 3000 to 5000). China had the highest DALYs (233,000, 95% UI 180,000 to 291,000), followed by the United States (215,000, 95% UI 176,000 to 256,000) and India (164,000, 95% UI 118,000 to 208,000).

In 2019, the countries with the highest age-standardized incidence rates were Canada (36.97, 95% UI 35.65 to 38.27 per 100,000 person-years), Norway (36.64, 95% UI 31.66 to 42.06 per 100,000 person-years), and Sweden (27.26, 95% UI 23.97 to 31.28 per 100,000 person-years). The countries with the lowest age-standardized incidence rates were Thailand (0.46, 95% UI 0.38 to 0.55 per 100,000 person-years), Cambodia (0.49, 95% UI 0.40 to 0.59 per 100,000 person-years), and Laos (0.51, 95% UI 0.42 to 0.63 per 100,000 person-years).

In 2019, the countries with the highest age-standardized mortality rates were the Netherlands (2.08, 95% UI 1.58 to 2.40 per 100,000 person-years), Germany (1.94, 95% UI 1.45 to 2.20 per 100,000 person-years), and Brunei (1.48, 95% UI 0.96 to 1.89 per 100,000 person-years). The countries with the lowest age-standardized mortality rates were Singapore (0.07, 95% UI 0.06 to 0.11 per 100,000 person-years), Sri Lanka (0.10, 95% UI 0.07 to 0.13 per 100,000 person-years), and Japan (0.11, 95% UI 0.09 to 0.16 per 100,000 person-years).

In 2019, the countries with the highest age-standardized DALY rates were Norway (80.37, 95% UI 56.25 to 108.56 per 100,000 person-years), Canada (64.91, 95% UI 46.59 to 85.11 per 100,000 person-years), and Hungary (64.34, 95% UI 47.69 to 82.54 per 100,000 person-years). The countries with the lowest age-standardized DALY rates were Thailand (3.64, 95% UI 2.76 to 4.68 per 100,000 person-years), Sri Lanka (3.77, 95% UI 2.86 to 4.79 per 100,000 person-years), and Myanmar (4.34, 95% UI 3.28 to 6.04 per 100,000 person-years).

From 1990 to 2019, there were significant differences in the EAPC of age-standardized incidence rates among different countries. Taiwan Province, Vietnam, and China had the highest increases, with EAPC values of 3.87, 2.62, and 2.54. On the other hand, Poland, the Netherlands, and the United States had the largest decreases, with EAPC values of − 3.85, − 1.79, and − 1.

In terms of age-standardized mortality rates from 1990 to 2019, different countries had varying EAPC values. Germany, Italy, and Serbia had the highest increases, with EAPC values of 3.73 (95% UI 3.26 to 4.20), 2.55 (95% UI 2.03 to 3.06), and 2.49 (95% UI 2.05 to 2.94), respectively. On the other hand, South Korea, Albania, and the Northern Mariana Islands had the largest decreases, with EAPC values of − 7.96 (95% UI − 8.67 to − 7.25), − 5.17 (95% UI − 6.14 to − 4.19), and − 4.76 (95% UI − 5.32 to − 4.19).

In terms of the EAPC values of age-standardized DALY rates, Oman, Argentina, and Yemen had the highest increases, with EAPC values of 1.88 (95% UI 1.53 to 2.23), 1.80 (95% UI 1.57 to 2.02), and 1.57 (95% UI 1.10 to 2.05). Conversely, Malta, Bolivia (Plurinational State of), and Montenegro had the largest decreases, with EAPC values of − 5.08 (95% UI − 5.69 to − 4.47), − 4.28 (95% UI − 4.78 to − 3.78), and − 4.23 (95% UI − 4.59 to − 3.86).

### Socio-demographic index

**Fig. 4:**
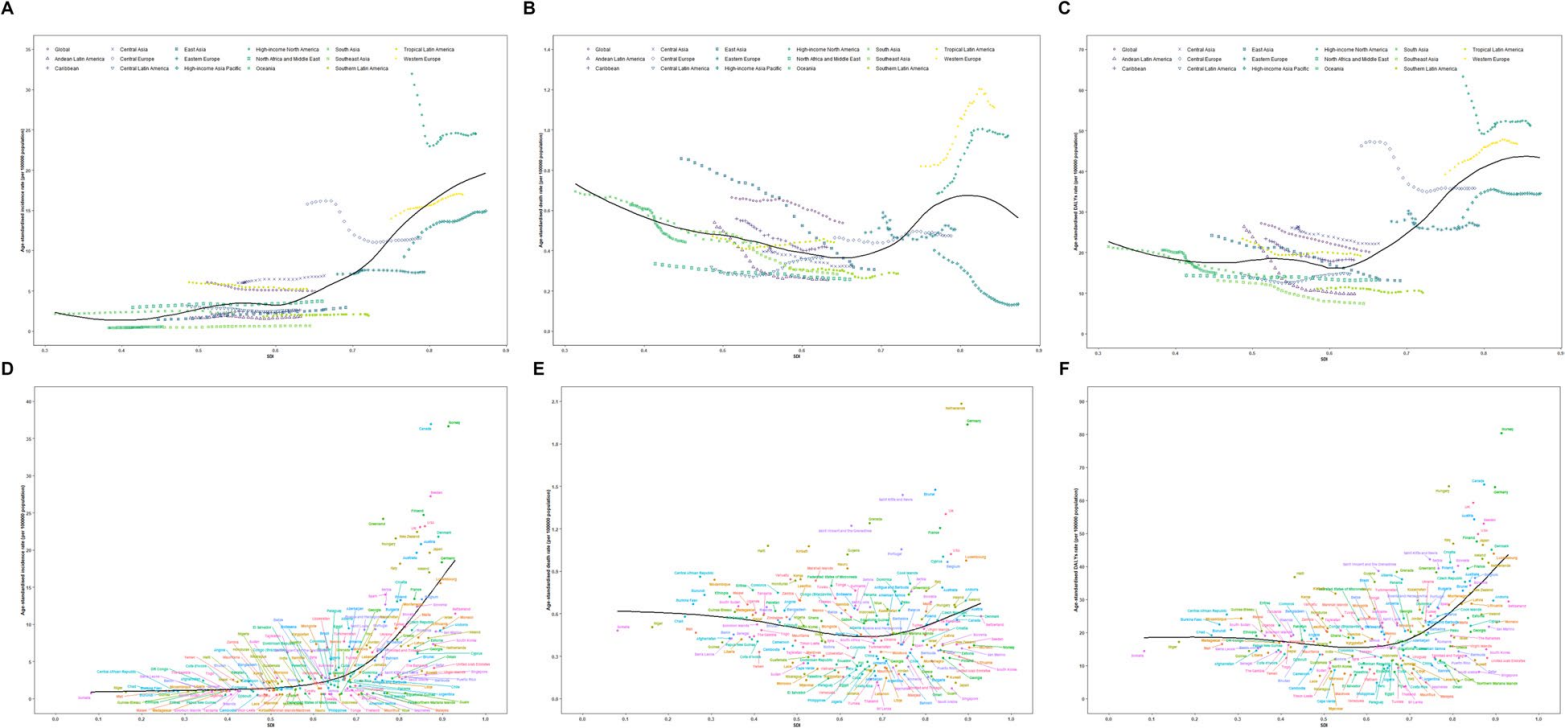
Inflammatory bowel disease for Global Burden of Disease region by SDI. Age-standardized incidence (**A**), death **(B**) and DALY (**C**) rates for Inflammatory bowel disease for 21 Global Burden of Disease region by SDI, 1990–2019. Age-standardized incidence (**D**), death (**E**) and DALY (**F**) rates for Inflammatory bowel disease for 198 countries and territories by SDI, 2019. Black line represents the expected age-standardized incidence (**A**), death (**B**) and DALY (**C**) rates of Inflammatory bowel disease based solely on SDI. For each region, points from the left to right depict estimates from each year from 1990 to 2019. SDI Sociodemographic Index, DALY disability adjusted life years

In regions with initially low SDI, such as South Asia and Oceania, age-standardized incidence rates continuously increase as SDI grows. In regions with moderate SDI, such as Central Latin America and tropical Latin America, age-standardized incidence rates decline with increasing SDI. In regions with initially high SDI, like high-income North America and Central Europe, where SDI values range from 0.7 to 0.9, incidence rates continue to decrease as SDI increases. However, with further increases in SDI, age-standardized incidence rates start to rise again (Fig. 4A).

In regions with moderate or lower initial SDI (except for Central Latin America and tropical Latin America), age-standardized mortality rates decrease as SDI increases. In regions with initially high SDI, age-standardized mortality rates increase with increasing SDI. However, in Western Europe and high-income North America, age-standardized mortality rates start to decrease again when SDI reaches around 0.82 (Fig. 4B).

Overall, age-standardized DALY rates reach their lowest point at an SDI value of approximately 0.61. As SDI increases, age-standardized DALY rates rise again. The decline in age-standardized DALY rates is more significant in East Asia and the Andean Latin America regions, while Western Europe and high-income North America experience a larger increase in age-standardized DALY rates (Fig. 4C).

Regarding national SDI, we conducted a comprehensive analysis of the average data of SDI, age-standardized incidence rates, age-standardized mortality rates, and age-standardized DALY rates for all countries. We simulated three curves: age-standardized incidence rates-SDI, age-standardized mortality rates-SDI, and age-standardized DALY rates-SDI. Overall, as the SDI of a country increases, its corresponding age-standardized incidence rates also rise. However, not all countries perfectly follow the expected pattern of the curve. For example, Canada and Norway have significantly higher age-standardized incidence rates than expected based on their SDI, while countries like South Africa and Indonesia have lower age-standardized incidence rates than expected (Fig. 4D).

Similarly, age-standardized mortality rates continuously decrease before a country’s SDI reaches 0.7 and then start to increase afterward (Fig. 4E). Age-standardized DALY rates follow a similar pattern, with a continuous decline until an SDI value of 0.65 and then a subsequent increase (Fig. 4F).

### Bayesian age-period-cohort model prediction

We conducted an APC analysis for IBD and predicted the number of new cases and deaths from IBD from 2019 to 2050. The shaded area represents the baseline reference values based on assuming a stable incidence rate observed in 2019, with an annual decrease of 1% (optimistic reference, lower bound) or an annual increase of 1% (pessimistic reference, upper bound).

**Fig. 5:**
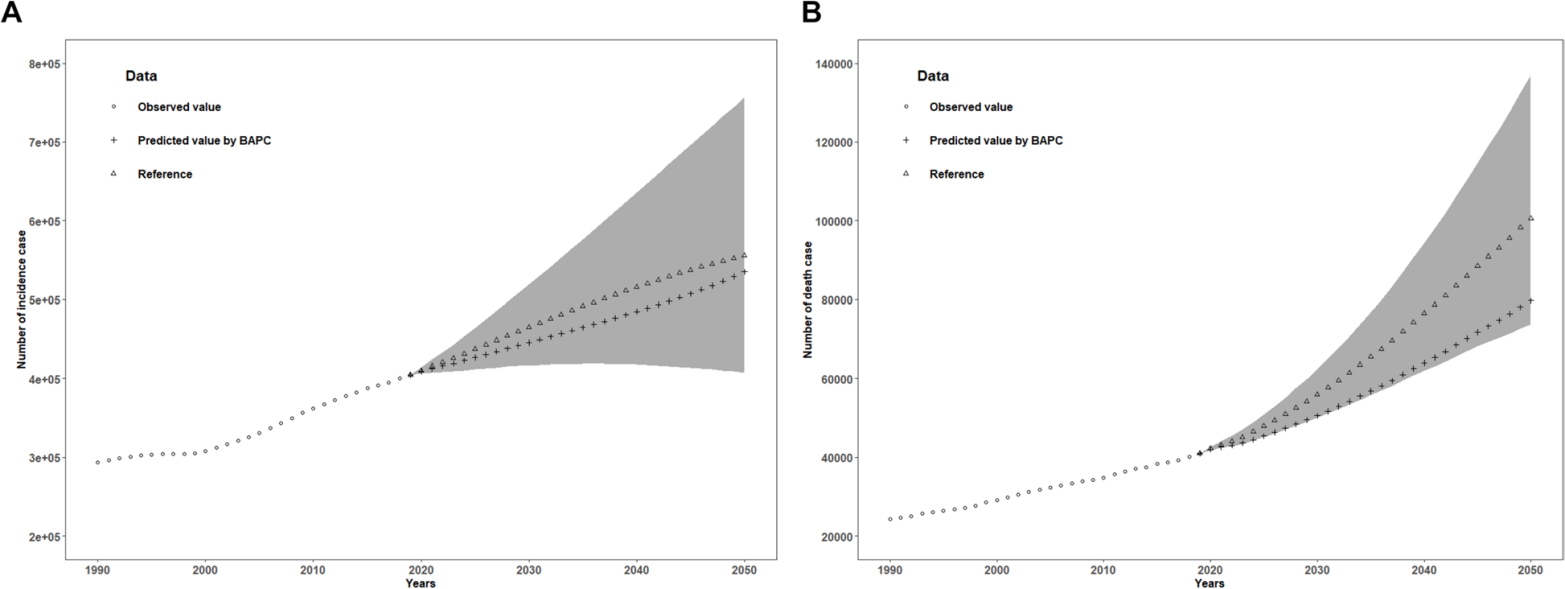
Predictions for inflammatory bowel disease in next 30 years. Trends in observed (“○” lines) and predicted (“+” lines) Inflammatory bowel disease in number of incidence cases (**A**) and deaths (**B**). Scalloped gray shading (“{” lines) indicates if the rate remained stable (baseline reference), decreased by 1% per year (optimistic reference, lower limit), and increased by 1% per year (pessimistic reference, upper limit) based on the observed rate in 2019(“△” lines)

The predicted number of new cases and deaths from IBD between 2019 and 2050 is lower than the reference values. However, the slope of the predicted incidence curve for new cases is almost parallel to the slope of the observed incidence curve based on 2019 data. This suggests that the age-standardized incidence rate of the population may remain relatively similar to the rate observed in 2019 over the next 30 years (Fig. 5A).

On the other hand, the slope of the predicted mortality curve is smaller than the slope of the mortality curve based on 2019 data. Therefore, the age-standardized mortality rate from IBD may continue to decline from 2020 to 2050 (Fig. 5B).

These predictions indicate that although the number of new cases and deaths from IBD is expected to be lower than the reference values, the age-standardized incidence rate may remain relatively stable, while the age-standardized mortality rate is likely to continue decreasing. It’s important to consider these predictions in the context of other factors that may influence the trends in IBD incidence and mortality, such as advancements in treatments and changes in risk factors over time.

## Discussion

In this study, we conducted a comprehensive analysis of the burden of IBD from 1990 to 2019 at the age and sex, global, regional, and national levels. From 1990 to 2019, the age-standardized incidence rate, age-standardized mortality rate, and age-standardized DALYs rate for IBD have all declined possibly due to effective implementation of global public prevention measures [14, 15]. However, due to the continuous growth of the population, the number of incidence, deaths, and DALYs has still increased each year.

From 1990 to 2000, the age-standardized incidence rate for both males and females rapidly declined, possibly due to the adoption of preventive measures such as dietary interventions, drug prophylaxis and vaccination and the saturation of genetic and environmental factors contributing to the development of IBD in some regions, such as Europe and North America [16], which may have reached a plateau in the incidence of IBD. Those may have reduced the age-standardized incidence rate of inflammatory bowel disease. From 2000 to 2019, the age-standardized incidence rate for both males and females continued to decline, but at a slower rate compared to the period of 1990 to 2000. During the period of 2000 to 2019, although the positive factors, the use of antibiotics and nonsteroidal anti-inflammatory drugs (NSAIDs) resulting from improvements in healthcare conditions, continued to improve, factors such as depression and social stress associated with social development, may lead to a slowdown in the reduction of the incidence of inflammatory bowel disease [17–19].

Furthermore, we observed an increase in age-standardized mortality rate among females at the end of the twentieth century, which may be associated with the increased proportion of female smokers, as smoking is a strong positive factor associated with disease progression in inflammatory bowel disease. Therefore, female IBD patients may benefit from smoking cessation [17, 18]. Since 2003, the age-standardized mortality rate for both males and females has decreased rapidly, which may be attributed to introduction of biological agents, colon cancer surveillance, and improved surgical techniques which collectively contributed to a decline in the mortality rate associated with IBD [20].

We observed that in 2019, there were two peaks in the incidence of IBD: one among individuals under the age of 35 and another among those aged 85 and above. The age group of 1–4 years had the highest mortality rate among individuals under the age of 35. Joannie Ruel et al. [21]. found that up to 25% of IBD cases occur during childhood or adolescence, while 10–15% of IBD patients are diagnosed after the age of 60. Additionally, the incidence of Crohn’s disease is higher in children compared to adults and older individuals. The characteristics of early-onset and elderly-onset Crohn’s disease predominantly involve isolated colonic disease, whereas in older children and adults, involvement of the ileocolon is more common [21]. In ulcerative colitis, the extensive disease pattern is more prevalent in early-onset cases compared to late-onset cases. Therefore, policymakers may need to allocate more healthcare resources to IBD patients in children, adolescents, and the elderly. Furthermore, extensive disease education is necessary to ensure early diagnosis and treatment for potential IBD patients, thereby preventing disease progression and the need for surgery.

In 2019, the highest age-standardized incidence rates were observed in high-income North American regions, Australasia, and Western Europe. The countries with the highest age-standardized incidence rates were Canada, Norway, and Sweden, which are all located in the aforementioned regions. Similar patterns were also observed in high SDI regions. Risk factors for IBD include excessive consumption of sugar, animal fats, and linoleic acid, while a high-fiber diet and consumption of citrus fruits may have protective effects [22]. According to Aaron S. Bancil et al. [23], food emulsifiers may contribute to the development of IBD through mechanisms such as promoting pro-inflammatory gut microbiota, disrupting mucus structure, increasing intestinal permeability, activating inflammatory pathways, and disrupting the cell cycle. An ecological analysis using national-level data showed a positive correlation between emulsifier consumption and the incidence of Crohn’s disease in Europe, North America, and Japan [24].

In other emerging industrialized regions and countries, such as East Asia (excluding high-income Asia-Pacific regions), the age-standardized incidence rates have continued to increase rapidly over the past 30 years. Based on the observed results from the BAPC model predictions, it is suggested that from 2019 to 2050, the incidence of IBD will not further increase, while the mortality rate will decrease.

The Bayesian model’s prediction from 2019 to 2050 of a decline in mortality while incidence case remains stable could be attributed to several factors:

Improved Healthcare Services: Over the years, advancements in healthcare services, including early detection and better management of IBD, could lead to a decline in mortality rates. The availability of better healthcare resources, effective treatment regimens, and improved patient management protocols might contribute to reducing the mortality associated with IBD while keeping the incidence rate stable. For example, advancements in healthcare that future medical and dietary therapies will adopt a microbiome-modulating approach to treatment [25].

Public Health Initiatives: Public health initiatives aimed at reducing the risk factors associated with IBD could also contribute to lowering mortality rates. These initiatives might include awareness campaigns, vaccination programs [26], and lifestyle modification recommendations that help in managing the disease better and reducing fatalities.

Technological Advancements: The integration of modern technologies in healthcare, such as Telemedicine, Artificial Intelligence, and Machine Learning, could enhance patient monitoring, disease management, and treatment personalization, thereby reducing mortality rates. For example, better management of IBD that encompass remote monitoring through Point Of Care (POC) testing, telehealth and teleconsultations, usage of mobile-based applications and wearables, implementing AI for endoscopy, and as a decision-making support tool. These measures can aid in reducing the burden on patients and healthcare providers [27].

Aging Population: The aging population might have developed better immunity or resistance to certain conditions associated with IBD [28], hence a decrease in mortality rates.

The prediction regarding incidence case remaining stable might indicate that while the aforementioned factors are effective in reducing mortality, they may not significantly impact the incidence case of IBD. The incidence case might remain stable due to persistent risk factors, genetic predispositions, or environmental factors that continue to contribute to the new cases of IBD.

In an article by Gilaad G. Kaplan et al. [29], four stages of global IBD development are mentioned, including the emergence phase, the accelerated incidence phase, the composite prevalence phase, and the endemic equilibrium phase. Currently, all countries and regions are in the first three stages. In the composite prevalence phase, under the influence of multiple factors, the incidence rate among the population is much higher than the mortality rate, leading to a significant increase in the number of individuals with IBD. As we approach the fourth stage, the endemic equilibrium phase, with the increasing population size and the proportion of the elderly population, the number of new cases among young individuals will gradually decrease. However, the elderly population is more prone to developing cancer and cardiovascular diseases, resulting in an increase in the overall mortality rate. As a result, the incidence rate may decrease, stabilize, or reach a balance, known as the endemic equilibrium phase [29–31]. This relative stability in the prevalence of IBD is of great importance for comprehensive decision-making on IBD healthcare provision at the national level.

There are limitations to this study. Firstly, although the GBD study provides estimates based on robust statistical methods, the lack of access to real data unavoidably introduces biases in our current research. Secondly, the data were collected by cancer-related departments in different countries, and there may be variations in their diagnostic criteria, leading to uncertainties. Thirdly, the relationship between IBD and certain risk factors should be considered in future studies.

Overall, there has been a decrease in the global incidence, mortality, and DALYs rates of IBD. However, due to the growing population, the number of new cases, deaths, and DALYs continues to rise, maintaining an upward trend for a significant period. This highlights the need for continued public health efforts to sustain and further improve the declining rates of incidece, mortality and DALYs, especially in the face of population growth. Moreover, emerging industrialized countries are experiencing an accelerating increase in incidence rates, gradually approaching those of developed countries like Europe and the United States. Policymakers in these countries need to enhance healthcare provisions to shorten the transition from a complex epidemic to a balanced one, thus alleviating the global burden of IBD.

## Conclusions

IBD has shown a consistent increase in global incidence, mortality, and DALYs in recent years. This study provides global IBD data and trends across regions with different socioeconomic statuses. It also predicts the future trends of IBD cases and deaths worldwide until 2050 based on current patterns. The findings contribute to the development of effective policies and care strategies for addressing IBD challenges in diverse regions and socioeconomic contexts.

### Supplementary Information

**Additional file 1.**

## Data Availability

The datasets generated during and/or analyzed during the current study are available from the Global Health Data Exchange query tool (http://ghdx.healthdata.org/gbd-results-tool).

## Abbreviations

IBD: inflammatory bowel disease
DALYs: disability-adjusted life years
EAPC: estimated annual percentage change
GBD: global burden of disease
SDI: socio-demographic index
UI: uncertainty intervals
ASR: age-standardized rates
YLDs: years lived with disability
YLLs: years of life lost
CI: confidence interval
BAPC: bayesian age-period-cohort
APC: age-period-cohort
NSAIDs: nonsteroidal anti-inflammatory drugs
Point Of Care: Point Of Care
