# Supplementary material for: Trends and projections of inflammatory bowel disease at the global, regional and national levels, 1990–2050: a bayesian age-period-cohort modeling study
Source: BMC Public Health. 2023 Dec 14;23:2507. doi: 10.1186/s12889-023-17431-8 (PMC10722679; doi:10.1186/s12889-023-17431-8)
Supplement: Supplementary file 1 — Additional file 1. [file 12889_2023_17431_MOESM1_ESM.docx]

**Table S1**. The rate of incidence, deaths, DALYs, ASIR, ASMR, ASDR for Inflammatory bowel disease in 2019 , EAPC and ASRs by Global Burden from 1990 to 2019

| location | 2019 | | 1990–2019 | 2019 | | 1990–2019 | 2019 | | 1990–2019 |
| --- | --- | --- | --- | --- | --- | --- | --- | --- | --- |
|  | Incidece rate per 100,000 population (95%UI) | Age Standardized Incidence Rate(ASIR) per 100,000 population (95%UI) | EAPC in ASIR  (95% UI) | Mortality rate per 100,000 population (95%UI) | Age Standardized Mortality Rate(ASMR) per 100,000 population (95%UI) | EAPC in ASMR  (95% UI) | DALYs rate per 100,000 population (95%UI) | Age Standardized DALY Rate(ASDR) per 100,000 population (95%UI) | EAPC in ASDR  (95% UI) |
| Global | 5.23 (4.66 to 5.9) | 4.97(4.43 to 5.59) | -0.60 (-0.73 to -0.48) | 0.53 (0.45 to 0.58) | 0.54(0.46 to 0.59) | -0.69 (-0.81 to -0.57) | 20.97 (17.53 to 24.75) | 20.15(16.86 to 23.71) | -1.04 (-1.06 to -1.01) |
| Afghanistan | 1.79 (1.46 to 2.16) | 2.45 (2.03 to 2.96) | 0.28 (0.16 to 0.40) | 0.15 (0.08 to 0.23) | 0.43 (0.26 to 0.64) | 0.01 (-0.06 to 0.08) | 7.88 (4.91 to 12.18) | 14.58 (9.58 to 20.47) | 0.03 (-0.06 to 0.12) |
| Albania | 10.68 (9.17 to 12.54) | 9.48 (8.09 to 11.13) | 0.68 (0.61 to 0.76) | 0.46 (0.31 to 0.7) | 0.41 (0.27 to 0.61) | -5.17 (-6.14 to -4.19) | 36.42 (26.73 to 47.8) | 36.18 (25.65 to 49.28) | -4.07 (-5.01 to -3.12) |
| Algeria | 3.96 (3.28 to 4.88) | 3.87 (3.23 to 4.74) | 0.95 (0.86 to 1.04) | 0.13 (0.09 to 0.17) | 0.17 (0.13 to 0.23) | -1.14 (-1.23 to -1.04) | 9.61 (7.16 to 12.65) | 9.93 (7.45 to 12.97) | -0.28 (-0.38 to -0.17) |
| American Samoa | 0.62 (0.52 to 0.74) | 0.64 (0.54 to 0.77) | 1.10 (0.97 to 1.22) | 0.42 (0.3 to 0.74) | 0.53 (0.37 to 0.9) | -4.40 (-4.80 to -4.01) | 14 (9.79 to 24.63) | 15.67 (10.89 to 28.11) | -4.23 (-4.59 to -3.86) |
| Andorra | 11.4 (9.7 to 13.56) | 9.18 (7.75 to 10.83) | 0.20 (0.09 to 0.32) | 1.27 (0.76 to 1.83) | 0.69 (0.41 to 1.01) | 0.03 (-0.06 to 0.13) | 43.79 (31.16 to 57.15) | 29.68 (20.92 to 39.03) | 0.02 (-0.03 to 0.07) |
| Angola | 0.87 (0.73 to 1.04) | 1.32 (1.12 to 1.59) | 0.71 (0.67 to 0.76) | 0.26 (0.17 to 0.39) | 0.62 (0.45 to 0.89) | -0.60 (-0.66 to -0.54) | 12.15 (7.46 to 19.2) | 18.19 (12.6 to 26.15) | -0.89 (-0.95 to -0.83) |
| Antigua and Barbuda | 3.02 (2.56 to 3.67) | 2.6 (2.21 to 3.13) | 0.56 (0.42 to 0.71) | 0.71 (0.55 to 0.97) | 0.67 (0.53 to 0.91) | -1.77 (-2.08 to -1.46) | 24.8 (19.88 to 32.03) | 22.35 (17.9 to 28.99) | -1.66 (-1.90 to -1.42) |
| Argentina | 1.84 (1.54 to 2.18) | 1.73 (1.44 to 2.03) | 0.12 (0.10 to 0.15) | 0.32 (0.28 to 0.37) | 0.27 (0.23 to 0.31) | -0.10 (-0.25 to 0.04) | 10.1 (8.65 to 11.91) | 9.02 (7.72 to 10.63) | -0.13 (-0.22 to -0.05) |
| Armenia | 7.84 (6.63 to 9.36) | 6.64 (5.62 to 7.95) | 0.71 (0.66 to 0.77) | 0.36 (0.27 to 0.44) | 0.28 (0.21 to 0.34) | 0.72 (0.36 to 1.09) | 25.3 (19.64 to 32.43) | 20.5 (15.81 to 26.35) | 0.20 (0.05 to 0.36) |
| Australia | 21.79 (19.4 to 24.5) | 19.62 (17.36 to 22.17) | 1.13 (0.85 to 1.41) | 1.5 (1.06 to 1.75) | 0.78 (0.56 to 0.91) | 2.23 (1.81 to 2.66) | 47.09 (37.08 to 58.35) | 35.58 (27.28 to 45.33) | 1.20 (0.98 to 1.41) |
| Austria | 21.75 (19.62 to 24.47) | 20.81 (18.8 to 23.22) | 0.45 (0.31 to 0.59) | 1.13 (0.9 to 1.85) | 0.52 (0.43 to 0.81) | -1.59 (-2.15 to -1.03) | 72.49 (53.12 to 93.83) | 54.31 (39.35 to 71.37) | -0.39 (-0.65 to -0.12) |
| Azerbaijan | 7.83 (6.59 to 9.39) | 7.01 (5.92 to 8.4) | 0.18 (0.13 to 0.23) | 0.13 (0.09 to 0.17) | 0.16 (0.11 to 0.23) | -1.09 (-1.19 to -1.00) | 17.8 (12.54 to 24.03) | 16.2 (11.65 to 21.65) | -0.36 (-0.44 to -0.27) |
| Bahamas | 3.67 (3.04 to 4.44) | 3.25 (2.71 to 3.92) | 1.14 (1.01 to 1.27) | 0.49 (0.39 to 0.63) | 0.49 (0.39 to 0.62) | -0.66 (-0.78 to -0.53) | 20.68 (16.61 to 25.98) | 19.04 (15.33 to 23.86) | -0.72 (-0.81 to -0.63) |
| Bahrain | 4.04 (3.31 to 4.98) | 3.21 (2.68 to 3.88) | 0.45 (0.28 to 0.62) | 0.08 (0.06 to 0.11) | 0.17 (0.13 to 0.22) | -1.34 (-1.69 to -0.99) | 11.56 (8.01 to 15.9) | 10.05 (7.4 to 13.28) | -0.53 (-0.63 to -0.43) |
| Bangladesh | 2.14 (1.75 to 2.6) | 2.17 (1.78 to 2.65) | 0.70 (0.62 to 0.78) | 0.45 (0.27 to 0.67) | 0.56 (0.35 to 0.83) | -2.14 (-2.39 to -1.89) | 16.55 (10.9 to 23.39) | 18.1 (12.11 to 25.27) | -2.03 (-2.15 to -1.92) |
| Barbados | 6.06 (5.15 to 7.34) | 5.13 (4.39 to 6.1) | 0.33 (0.17 to 0.50) | 0.59 (0.46 to 0.75) | 0.38 (0.3 to 0.48) | -0.41 (-0.57 to -0.25) | 27.11 (20.96 to 34.08) | 20.02 (15.56 to 25.32) | -0.42 (-0.48 to -0.36) |
| Belarus | 6.84 (5.81 to 8.15) | 5.71 (4.86 to 6.75) | 0.71 (0.65 to 0.77) | 0.61 (0.44 to 0.81) | 0.38 (0.28 to 0.5) | 0.24 (0.09 to 0.39) | 32.24 (24.88 to 41.29) | 23.25 (17.89 to 29.87) | 0.17 (0.09 to 0.25) |
| Belgium | 14.46 (12.7 to 16.8) | 13.4 (11.65 to 15.72) | 1.96 (1.56 to 2.37) | 2.39 (1.8 to 2.79) | 0.97 (0.76 to 1.12) | 0.24 (-0.90 to 1.40) | 55.78 (45.85 to 67.24) | 36.56 (28.88 to 45.93) | 0.82 (0.35 to 1.28) |
| Belize | 2.52 (2.1 to 3.06) | 2.7 (2.26 to 3.27) | 0.56 (0.43 to 0.68) | 0.44 (0.36 to 0.58) | 0.61 (0.5 to 0.8) | -1.14 (-1.52 to -0.76) | 18.88 (15.53 to 23.74) | 21.8 (18.03 to 27.26) | -1.22 (-1.49 to -0.95) |
| Benin | 0.76 (0.63 to 0.91) | 1.16 (0.98 to 1.39) | 0.33 (0.23 to 0.44) | 0.23 (0.14 to 0.35) | 0.45 (0.3 to 0.65) | -0.42 (-0.64 to -0.20) | 11.35 (6.11 to 18.01) | 15.67 (9.98 to 22.61) | -0.50 (-0.70 to -0.30) |
| Bermuda | 3.85 (3.24 to 4.65) | 2.91 (2.46 to 3.47) | 0.54 (0.40 to 0.69) | 0.7 (0.54 to 0.96) | 0.36 (0.28 to 0.5) | -3.73 (-4.07 to -3.40) | 21.7 (17.16 to 27.96) | 14.3 (11.3 to 18.41) | -2.73 (-3.00 to -2.46) |
| Bhutan | 1.87 (1.55 to 2.3) | 1.86 (1.55 to 2.29) | 0.09 (-0.11 to 0.29) | 0.38 (0.23 to 0.87) | 0.55 (0.34 to 1.23) | -0.84 (-0.97 to -0.72) | 13.49 (8.84 to 26.77) | 15.82 (10.39 to 31.52) | -1.18 (-1.27 to -1.10) |
| Bolivia (Plurinational State of) | 1.64 (1.39 to 1.97) | 1.85 (1.58 to 2.23) | -0.17 (-0.38 to 0.04) | 0.29 (0.21 to 0.37) | 0.4 (0.3 to 0.52) | -0.83 (-0.91 to -0.75) | 11.23 (8.21 to 14.75) | 12.73 (9.53 to 16.38) | -2.04 (-2.15 to -1.94) |
| Bosnia and Herzegovina | 11.07 (9.85 to 12.64) | 9.26 (8.25 to 10.53) | 1.12 (0.97 to 1.26) | 0.78 (0.39 to 1.07) | 0.45 (0.23 to 0.61) | -0.20 (-0.52 to 0.12) | 41.57 (28.74 to 53.88) | 29.74 (21.21 to 38.84) | 0.14 (-0.01 to 0.28) |
| Botswana | 1.34 (1.11 to 1.65) | 1.47 (1.22 to 1.79) | 0.62 (0.52 to 0.72) | 0.38 (0.25 to 0.55) | 0.64 (0.43 to 0.89) | -0.77 (-1.15 to -0.39) | 16.59 (10.32 to 26.5) | 20.3 (13.14 to 30.81) | -0.41 (-0.71 to -0.11) |
| Brazil | 5.86 (5.14 to 6.75) | 5.3 (4.65 to 6.09) | -0.54 (-0.59 to -0.50) | 0.48 (0.43 to 0.56) | 0.45 (0.41 to 0.52) | 0.01 (-0.16 to 0.19) | 21.39 (18.3 to 25.01) | 19.51 (16.72 to 22.74) | -0.44 (-0.62 to -0.27) |
| Brunei Darussalam | 5.89 (4.75 to 7.06) | 5.09 (4.17 to 6.09) | 0.08 (0.04 to 0.12) | 0.59 (0.48 to 0.74) | 1.48 (0.97 to 1.89) | 0.09 (-0.02 to 0.20) | 26.05 (20.26 to 33.62) | 32.76 (26.62 to 39.71) | -0.25 (-0.33 to -0.18) |
| Bulgaria | 12.27 (10.52 to 14.36) | 10.34 (8.83 to 12.16) | 0.76 (0.53 to 0.99) | 0.59 (0.46 to 0.74) | 0.33 (0.25 to 0.41) | 1.13 (0.73 to 1.54) | 37.05 (27.94 to 47.86) | 27.62 (20.56 to 35.57) | 0.00 (-0.21 to 0.21) |
| Burkina Faso | 0.7 (0.58 to 0.86) | 1.05 (0.89 to 1.27) | 0.21 (0.06 to 0.36) | 0.36 (0.18 to 0.57) | 0.67 (0.38 to 1) | 0.56 (0.28 to 0.85) | 17.37 (7.62 to 30.87) | 22.52 (12.05 to 34.8) | 0.38 (0.15 to 0.62) |
| Burundi | 0.66 (0.54 to 0.8) | 0.97 (0.81 to 1.18) | 0.27 (0.22 to 0.32) | 0.32 (0.2 to 0.49) | 0.73 (0.46 to 1.16) | 0.15 (-0.07 to 0.37) | 13.47 (8.39 to 21.18) | 20.85 (13.56 to 32.18) | -0.20 (-0.42 to 0.03) |
| Cabo Verde | 1.14 (0.95 to 1.37) | 1.19 (1 to 1.44) | 0.68 (0.60 to 0.77) | 0.2 (0.15 to 0.25) | 0.25 (0.19 to 0.33) | -2.05 (-2.56 to -1.54) | 7.68 (6.14 to 9.56) | 8.69 (6.98 to 10.77) | -2.12 (-2.50 to -1.74) |
| Cambodia | 0.48 (0.39 to 0.58) | 0.49 (0.4 to 0.59) | 1.06 (1.01 to 1.12) | 0.23 (0.16 to 0.29) | 0.35 (0.23 to 0.45) | -1.44 (-1.54 to -1.34) | 7.36 (5.36 to 9.31) | 8.98 (6.42 to 11.33) | -1.86 (-1.96 to -1.77) |
| Cameroon | 0.91 (0.75 to 1.11) | 1.28 (1.08 to 1.56) | 0.21 (0.05 to 0.37) | 0.2 (0.1 to 0.33) | 0.38 (0.21 to 0.59) | -1.69 (-1.94 to -1.43) | 9.72 (5.22 to 15.94) | 13.36 (7.77 to 21.03) | -1.43 (-1.66 to -1.20) |
| Canada | 38.9 (37.5 to 40.22) | 36.97 (35.65 to 38.27) | 0.28 (0.12 to 0.44) | 1.1 (0.88 to 1.66) | 0.56 (0.46 to 0.82) | -2.43 (-3.11 to -1.75) | 81.92 (59.98 to 105.26) | 64.91 (46.59 to 85.11) | -0.31 (-0.41 to -0.21) |
| Central African Republic | 0.8 (0.66 to 0.96) | 1.13 (0.94 to 1.35) | 0.12 (0.10 to 0.14) | 0.39 (0.22 to 0.66) | 0.86 (0.53 to 1.36) | 0.02 (-0.06 to 0.10) | 16.79 (8.95 to 29.59) | 25.47 (14.87 to 41.5) | -0.07 (-0.13 to 0.00) |
| Chad | 0.59 (0.49 to 0.71) | 0.99 (0.83 to 1.2) | 0.18 (0.03 to 0.33) | 0.27 (0.16 to 0.39) | 0.58 (0.39 to 0.84) | -0.24 (-0.45 to -0.04) | 12.71 (6.22 to 21.1) | 19.16 (11.97 to 27.44) | -0.36 (-0.55 to -0.16) |
| Chile | 2.67 (2.31 to 3.09) | 2.44 (2.1 to 2.81) | 0.43 (0.20 to 0.66) | 0.39 (0.33 to 0.49) | 0.3 (0.26 to 0.38) | -1.75 (-2.10 to -1.40) | 14.35 (11.92 to 17.41) | 11.96 (9.91 to 14.48) | -1.10 (-1.28 to -0.91) |
| China | 3.62 (3.09 to 4.25) | 3.01 (2.59 to 3.5) | 2.54 (2.39 to 2.69) | 0.33 (0.27 to 0.38) | 0.3 (0.24 to 0.35) | -4.11 (-4.50 to -3.71) | 16.34 (12.65 to 20.47) | 13.1 (10.29 to 16.31) | -2.36 (-2.54 to -2.18) |
| Colombia | 2.36 (2.01 to 2.78) | 2.2 (1.86 to 2.59) | -0.17 (-0.34 to 0.01) | 0.32 (0.24 to 0.42) | 0.29 (0.22 to 0.38) | 0.75 (0.38 to 1.12) | 13.44 (10.45 to 16.72) | 12.53 (9.75 to 15.57) | 0.36 (0.12 to 0.60) |
| Comoros | 1 (0.82 to 1.21) | 1.13 (0.94 to 1.37) | 0.36 (0.28 to 0.44) | 0.51 (0.32 to 0.81) | 0.75 (0.48 to 1.19) | -0.51 (-0.67 to -0.36) | 16.64 (10.5 to 24.41) | 20.58 (13.1 to 30.54) | -0.63 (-0.84 to -0.41) |
| Congo | 1.14 (0.95 to 1.37) | 1.41 (1.18 to 1.68) | 0.81 (0.70 to 0.93) | 0.32 (0.23 to 0.47) | 0.66 (0.47 to 0.93) | -0.58 (-0.75 to -0.41) | 12.94 (8.8 to 18.6) | 18.36 (13.15 to 25.9) | -0.82 (-0.99 to -0.66) |
| Cook Islands | 0.83 (0.7 to 1) | 0.74 (0.63 to 0.9) | 1.18 (1.06 to 1.31) | 1 (0.66 to 1.38) | 0.81 (0.53 to 1.11) | -2.70 (-2.90 to -2.50) | 28.26 (18.1 to 39.69) | 24.94 (15.58 to 36.18) | -2.70 (-2.88 to -2.51) |
| Costa Rica | 2.54 (2.17 to 3) | 2.32 (1.99 to 2.73) | -0.46 (-0.59 to -0.33) | 0.21 (0.16 to 0.28) | 0.2 (0.14 to 0.26) | 1.04 (0.82 to 1.26) | 11.12 (8.45 to 14.18) | 10.14 (7.74 to 12.91) | 0.58 (0.47 to 0.69) |
| Croatia | 13.92 (12.81 to 15.19) | 13.09 (12.06 to 14.21) | 0.31 (0.19 to 0.43) | 0.21 (0.12 to 0.3) | 0.41 (0.3 to 0.52) | -0.71 (-0.97 to -0.45) | 53.86 (40.43 to 68.21) | 41.3 (30.43 to 53.17) | 0.79 (0.65 to 0.93) |
| Cuba | 3.16 (2.66 to 3.79) | 2.53 (2.12 to 3.02) | 0.93 (0.89 to 0.96) | 0.78 (0.6 to 1) | 0.23 (0.18 to 0.29) | 1.79 (1.42 to 2.17) | 14.81 (11.75 to 18.36) | 10.45 (8.37 to 12.94) | -1.38 (-1.64 to -1.12) |
| Cyprus | 6.97 (6.28 to 7.72) | 6.07 (5.48 to 6.74) | 0.35 (0.19 to 0.52) | 0.37 (0.29 to 0.47) | 1 (0.76 to 1.32) | -2.04 (-2.48 to -1.60) | 28.2 (23.04 to 33.83) | 22.06 (17.95 to 26.41) | -2.26 (-2.48 to -2.03) |
| Czechia | 9.78 (9.15 to 10.47) | 9.26 (8.6 to 9.97) | 1.22 (0.91 to 1.53) | 1.2 (0.94 to 1.53) | 0.56 (0.42 to 0.69) | -3.13 (-3.45 to -2.81) | 49.41 (38.77 to 61.98) | 35.4 (27.15 to 44.74) | 0.98 (0.90 to 1.06) |
| Côte d'Ivoire | 0.92 (0.76 to 1.14) | 1.26 (1.05 to 1.55) | 1.84 (1.64 to 2.04) | 1.07 (0.81 to 1.33) | 0.4 (0.26 to 0.58) | 0.80 (0.45 to 1.15) | 9.87 (5.73 to 15.1) | 13.59 (8.56 to 19.48) | -0.63 (-0.86 to -0.40) |
| Democratic People's Republic of Korea | 1.48 (1.24 to 1.76) | 1.29 (1.08 to 1.53) | 1.23 (1.07 to 1.39) | 0.41 (0.29 to 0.56) | 0.41 (0.28 to 0.59) | -1.10 (-1.20 to -1.00) | 12.05 (8.85 to 15.7) | 11.1 (8.17 to 14.58) | -1.31 (-1.41 to -1.21) |
| Democratic Republic of the Congo | 0.85 (0.7 to 1.04) | 1.22 (1.03 to 1.49) | 0.60 (0.52 to 0.68) | 0.26 (0.18 to 0.38) | 0.57 (0.4 to 0.82) | -0.78 (-0.91 to -0.64) | 11.43 (7.69 to 17.53) | 17.22 (12.2 to 24.09) | -0.82 (-0.97 to -0.66) |
| Denmark | 22.98 (21.5 to 24.46) | 21.82 (20.43 to 23.28) | -0.01 (-0.12 to 0.11) | 1.21 (1.03 to 1.42) | 0.59 (0.5 to 0.68) | 0.01 (-0.61 to 0.63) | 58.43 (45.57 to 73.04) | 45.17 (34.12 to 58.06) | 0.13 (-0.02 to 0.29) |
| Djibouti | 0.87 (0.71 to 1.05) | 1.01 (0.85 to 1.22) | -0.02 (-0.16 to 0.11) | 0.27 (0.17 to 0.45) | 0.54 (0.36 to 0.89) | -0.56 (-0.67 to -0.45) | 12 (7.29 to 19.59) | 15.83 (10.31 to 24.84) | -0.67 (-0.82 to -0.53) |
| Dominica | 3.39 (2.85 to 4.1) | 3.02 (2.54 to 3.62) | 1.13 (0.92 to 1.33) | 0.94 (0.71 to 1.27) | 0.76 (0.57 to 1.02) | -1.20 (-1.28 to -1.12) | 29.91 (23.16 to 39.7) | 26 (20.19 to 34.33) | -1.17 (-1.27 to -1.07) |
| Dominican Republic | 2.65 (2.21 to 3.21) | 2.68 (2.25 to 3.25) | 0.77 (0.64 to 0.89) | 0.21 (0.14 to 0.28) | 0.24 (0.17 to 0.32) | 0.09 (-0.13 to 0.32) | 10.19 (7.54 to 13.32) | 10.73 (7.96 to 13.99) | -0.27 (-0.50 to -0.04) |
| Ecuador | 1.84 (1.62 to 2.07) | 1.89 (1.67 to 2.13) | -0.07 (-0.42 to 0.28) | 0.19 (0.14 to 0.25) | 0.23 (0.17 to 0.3) | 0.55 (-0.03 to 1.13) | 8.39 (6.37 to 10.36) | 9.02 (6.84 to 11.09) | -0.33 (-0.74 to 0.08) |
| Egypt | 3.87 (3.2 to 4.73) | 4.23 (3.51 to 5.16) | 0.32 (0.17 to 0.47) | 0.12 (0.07 to 0.19) | 0.21 (0.12 to 0.35) | 0.32 (0.07 to 0.56) | 9.54 (6.77 to 12.73) | 11.49 (8.21 to 15.51) | 0.19 (0.10 to 0.27) |
| El Salvador | 2.37 (2.02 to 2.76) | 2.41 (2.06 to 2.81) | -0.22 (-0.36 to -0.09) | 0.14 (0.11 to 0.19) | 0.14 (0.11 to 0.2) | -3.87 (-4.22 to -3.52) | 6.99 (5.26 to 9.17) | 7.25 (5.45 to 9.54) | -2.87 (-3.18 to -2.56) |
| Equatorial Guinea | 1.09 (0.9 to 1.32) | 1.6 (1.36 to 1.92) | 1.92 (1.78 to 2.07) | 0.2 (0.12 to 0.31) | 0.54 (0.33 to 0.86) | -1.45 (-1.60 to -1.30) | 8.35 (5.14 to 12.97) | 14.77 (9.26 to 22.66) | -1.89 (-2.04 to -1.73) |
| Eritrea | 0.81 (0.66 to 0.97) | 1.09 (0.9 to 1.31) | 0.62 (0.57 to 0.67) | 0.34 (0.22 to 0.5) | 0.77 (0.52 to 1.12) | 0.71 (0.52 to 0.89) | 15.16 (9.61 to 24.48) | 22.27 (15.26 to 32.3) | 0.34 (0.20 to 0.49) |
| Estonia | 8 (7.14 to 9.01) | 6.82 (6.04 to 7.64) | 1.82 (1.65 to 1.99) | 0.65 (0.49 to 0.88) | 0.34 (0.26 to 0.47) | -2.35 (-2.68 to -2.02) | 34.12 (25.97 to 43.65) | 24.5 (18.49 to 31.62) | -1.07 (-1.32 to -0.81) |
| Eswatini | 1.18 (0.98 to 1.42) | 1.49 (1.26 to 1.81) | 0.48 (0.44 to 0.52) | 0.31 (0.21 to 0.45) | 0.6 (0.42 to 0.83) | 0.53 (0.14 to 0.91) | 13.36 (8.87 to 19.89) | 17.93 (12.43 to 25.37) | 0.38 (0.14 to 0.63) |
| Ethiopia | 0.66 (0.55 to 0.8) | 0.99 (0.84 to 1.2) | 0.64 (0.55 to 0.73) | 0.3 (0.18 to 0.45) | 0.72 (0.42 to 1.07) | -0.67 (-0.82 to -0.52) | 11.96 (7.83 to 18.51) | 19.04 (11.93 to 28.1) | -1.33 (-1.48 to -1.18) |
| Fiji | 0.71 (0.6 to 0.86) | 0.72 (0.61 to 0.86) | 1.01 (0.92 to 1.09) | 0.2 (0.14 to 0.26) | 0.26 (0.19 to 0.34) | -1.16 (-1.28 to -1.03) | 7.48 (5.42 to 10) | 8.17 (5.94 to 10.77) | -1.08 (-1.21 to -0.95) |
| Finland | 27.1 (24.31 to 29.85) | 24.74 (22.08 to 27.29) | 1.58 (1.37 to 1.79) | 0.71 (0.6 to 0.83) | 0.29 (0.25 to 0.34) | -2.22 (-2.47 to -1.96) | 62.62 (45.48 to 82.57) | 47.57 (33.68 to 63.99) | 1.06 (0.98 to 1.14) |
| France | 12.73 (12.04 to 13.31) | 13.45 (12.64 to 14.08) | 0.72 (0.65 to 0.80) | 3.29 (1.89 to 3.95) | 1.21 (0.73 to 1.42) | 0.74 (0.46 to 1.02) | 61.71 (45.44 to 72.94) | 39.44 (31.13 to 48.47) | 1.07 (0.94 to 1.20) |
| Gabon | 1.33 (1.1 to 1.62) | 1.56 (1.3 to 1.88) | 0.85 (0.82 to 0.89) | 0.31 (0.21 to 0.44) | 0.53 (0.38 to 0.76) | -0.49 (-0.70 to -0.28) | 11.69 (7.83 to 16.77) | 15.27 (10.62 to 21.51) | -0.71 (-0.88 to -0.54) |
| Gambia | 0.86 (0.71 to 1.05) | 1.22 (1.03 to 1.49) | 1.15 (1.07 to 1.23) | 0.23 (0.15 to 0.31) | 0.44 (0.29 to 0.6) | -1.52 (-1.81 to -1.24) | 9.57 (6.23 to 13.26) | 14.25 (9.59 to 19.1) | -1.56 (-1.85 to -1.28) |
| Georgia | 7.52 (6.37 to 8.91) | 6.32 (5.38 to 7.44) | -0.16 (-0.21 to -0.11) | 0.46 (0.29 to 0.59) | 0.29 (0.19 to 0.37) | 0.18 (-1.12 to 1.50) | 27.03 (20.23 to 34.63) | 20.25 (15.35 to 26.28) | 0.02 (-0.38 to 0.41) |
| Germany | 19.72 (17.44 to 22.32) | 18.37 (16.07 to 20.92) | 1.14 (0.86 to 1.41) | 5.05 (3.72 to 5.77) | 1.94 (1.46 to 2.2) | 3.73 (3.26 to 4.20) | 108.35 (87.99 to 126.86) | 64.06 (51.49 to 77.71) | 1.80 (1.57 to 2.02) |
| Ghana | 0.97 (0.81 to 1.17) | 1.2 (1.01 to 1.46) | 0.43 (0.28 to 0.58) | 0.33 (0.18 to 0.49) | 0.55 (0.31 to 0.82) | -0.97 (-1.23 to -0.70) | 14.29 (8.1 to 21.1) | 18.5 (10.85 to 26.95) | -1.02 (-1.27 to -0.77) |
| Greece | 8.82 (8.21 to 9.43) | 7.95 (7.36 to 8.56) | -0.09 (-0.17 to -0.01) | 0.64 (0.53 to 0.78) | 0.23 (0.19 to 0.27) | -0.36 (-0.92 to 0.20) | 23.45 (18.49 to 29.28) | 15.88 (11.92 to 20.55) | -0.78 (-0.85 to -0.71) |
| Greenland | 27.46 (23.85 to 31.72) | 24.18 (20.79 to 27.92) | 0.72 (0.59 to 0.84) | 0.74 (0.41 to 0.96) | 0.74 (0.38 to 0.96) | 1.07 (0.80 to 1.34) | 44.91 (33.1 to 58.59) | 38.3 (28.36 to 50.15) | 0.36 (0.24 to 0.47) |
| Grenada | 3.29 (2.73 to 4) | 2.98 (2.48 to 3.61) | 0.73 (0.57 to 0.88) | 1.27 (1.06 to 1.57) | 1.24 (1.03 to 1.52) | -0.55 (-0.68 to -0.41) | 41.43 (34.88 to 50.61) | 38.43 (32.58 to 46.61) | -0.64 (-0.73 to -0.56) |
| Guam | 0.78 (0.66 to 0.93) | 0.75 (0.64 to 0.9) | 1.19 (1.08 to 1.31) | 0.16 (0.12 to 0.24) | 0.16 (0.12 to 0.23) | -3.00 (-3.55 to -2.45) | 5.51 (4.2 to 7.8) | 5.39 (4.11 to 7.68) | -2.12 (-2.65 to -1.59) |
| Guatemala | 2.02 (1.7 to 2.38) | 2.33 (1.99 to 2.74) | -0.18 (-0.31 to -0.05) | 0.2 (0.15 to 0.26) | 0.29 (0.22 to 0.37) | 1.32 (0.72 to 1.92) | 9.91 (7.77 to 12.43) | 11.99 (9.52 to 14.91) | 0.71 (0.18 to 1.24) |
| Guinea | 0.72 (0.59 to 0.87) | 1.06 (0.89 to 1.27) | 0.16 (0.07 to 0.26) | 0.23 (0.11 to 0.36) | 0.4 (0.21 to 0.6) | -0.36 (-0.50 to -0.22) | 11.19 (4.82 to 19.33) | 14.41 (7.36 to 21.95) | -0.58 (-0.70 to -0.47) |
| Guinea-Bissau | 0.79 (0.64 to 0.96) | 1.13 (0.94 to 1.38) | 0.31 (0.21 to 0.41) | 0.3 (0.18 to 0.44) | 0.61 (0.4 to 0.85) | -0.81 (-0.93 to -0.68) | 13.92 (8 to 20.62) | 20.54 (12.76 to 28.9) | -0.94 (-1.07 to -0.80) |
| Guyana | 2.84 (2.38 to 3.42) | 2.87 (2.41 to 3.44) | 0.47 (0.30 to 0.65) | 0.84 (0.61 to 1.11) | 1.02 (0.76 to 1.33) | -0.53 (-0.70 to -0.35) | 33.32 (24.75 to 43.59) | 35.43 (26.51 to 46.07) | -0.46 (-0.59 to -0.32) |
| Haiti | 2.12 (1.76 to 2.61) | 2.42 (2.03 to 2.97) | 0.49 (0.33 to 0.65) | 0.69 (0.37 to 1.01) | 1.08 (0.61 to 1.59) | -0.77 (-0.89 to -0.65) | 30.5 (15.12 to 49.22) | 36.86 (19.94 to 54.22) | -0.94 (-1.12 to -0.77) |
| Honduras | 1.86 (1.56 to 2.19) | 2.16 (1.83 to 2.55) | -0.35 (-0.50 to -0.21) | 0.51 (0.31 to 0.7) | 0.83 (0.51 to 1.15) | 0.87 (0.73 to 1.02) | 18.5 (12.14 to 25.56) | 24.37 (15.88 to 32.67) | -0.38 (-0.43 to -0.32) |
| Hungary | 23.45 (22.33 to 24.64) | 21.55 (20.45 to 22.74) | 2.22 (1.94 to 2.51) | 1.04 (0.83 to 1.32) | 0.56 (0.45 to 0.71) | -0.62 (-1.04 to -0.20) | 86.2 (64.67 to 108.99) | 64.34 (47.69 to 82.55) | 1.15 (0.85 to 1.46) |
| Iceland | 18.75 (16.25 to 21.18) | 17.01 (14.81 to 19.23) | 0.84 (0.54 to 1.14) | 1.19 (0.91 to 1.41) | 0.65 (0.51 to 0.75) | 0.14 (-0.01 to 0.29) | 54.09 (41.1 to 69.16) | 42.38 (31.83 to 54.83) | 0.47 (0.33 to 0.62) |
| India | 2.28 (1.9 to 2.8) | 2.34 (1.95 to 2.86) | 0.42 (0.24 to 0.62) | 0.3 (0.21 to 0.4) | 0.4 (0.29 to 0.53) | -1.93 (-2.11 to -1.74) | 11.82 (8.45 to 14.98) | 13.04 (9.44 to 16.5) | -1.73 (-1.86 to -1.61) |
| Indonesia | 0.58 (0.48 to 0.7) | 0.55 (0.46 to 0.66) | 0.85 (0.76 to 0.93) | 0.35 (0.22 to 0.45) | 0.55 (0.33 to 0.7) | -1.42 (-1.56 to -1.29) | 9.61 (7.04 to 12.43) | 11.66 (8.16 to 14.98) | -2.04 (-2.17 to -1.91) |
| Iran (Islamic Republic of) | 3.76 (3.08 to 4.62) | 3.43 (2.86 to 4.23) | 0.21 (0.06 to 0.36) | 0.16 (0.06 to 0.19) | 0.19 (0.08 to 0.24) | -0.11 (-0.64 to 0.42) | 11.25 (7.92 to 14.76) | 11.03 (7.6 to 14.22) | 0.12 (-0.02 to 0.25) |
| Iraq | 2.34 (1.92 to 2.83) | 2.54 (2.12 to 3.08) | 1.33 (1.19 to 1.46) | 0.12 (0.09 to 0.16) | 0.19 (0.15 to 0.27) | -1.58 (-1.76 to -1.39) | 8.83 (6.67 to 11.71) | 10.42 (8.08 to 13.32) | -0.96 (-1.14 to -0.78) |
| Ireland | 8.9 (7.78 to 10.26) | 8.17 (7.14 to 9.44) | -0.76 (-0.97 to -0.55) | 1.03 (0.83 to 1.31) | 0.66 (0.53 to 0.84) | -1.61 (-2.56 to -0.66) | 33.06 (26.36 to 41.4) | 25.5 (20.1 to 32.09) | -0.80 (-1.30 to -0.30) |
| Israel | 7 (6.43 to 7.75) | 7.07 (6.48 to 7.84) | 0.33 (0.14 to 0.51) | 0.49 (0.41 to 0.68) | 0.38 (0.32 to 0.51) | -1.01 (-1.36 to -0.66) | 19.88 (15.95 to 24.77) | 18.5 (14.69 to 23.2) | -0.65 (-0.76 to -0.54) |
| Italy | 20.22 (17.84 to 23.02) | 18.16 (16.07 to 20.44) | 0.33 (0.17 to 0.49) | 2.16 (1.56 to 2.43) | 0.76 (0.55 to 0.85) | 2.55 (2.03 to 3.06) | 73.66 (57.42 to 90.88) | 46.95 (35.41 to 59.78) | 0.17 (0.00 to 0.34) |
| Jamaica | 2.69 (2.26 to 3.22) | 2.5 (2.11 to 2.99) | 0.72 (0.54 to 0.90) | 0.26 (0.19 to 0.36) | 0.24 (0.17 to 0.33) | -1.45 (-1.78 to -1.12) | 12.2 (9.39 to 15.35) | 11.5 (8.84 to 14.5) | -1.28 (-1.50 to -1.06) |
| Japan | 17.56 (15.14 to 20.27) | 19.65 (16.87 to 22.71) | 1.32 (1.02 to 1.61) | 0.35 (0.27 to 0.49) | 0.11 (0.09 to 0.16) | -2.39 (-2.83 to -1.95) | 52.76 (36.68 to 71.6) | 46.58 (31.41 to 63.54) | 1.21 (0.88 to 1.54) |
| Jordan | 6.69 (5.58 to 7.88) | 6.91 (5.78 to 8.15) | 1.49 (1.33 to 1.66) | 0.12 (0.09 to 0.15) | 0.23 (0.18 to 0.3) | -2.62 (-2.98 to -2.26) | 18.52 (13.2 to 25.38) | 22.01 (16.06 to 29.65) | 0.46 (0.34 to 0.58) |
| Kazakhstan | 7.55 (6.45 to 9.03) | 7.2 (6.14 to 8.56) | 0.43 (0.30 to 0.56) | 0.44 (0.36 to 0.54) | 0.46 (0.37 to 0.56) | -1.26 (-1.68 to -0.85) | 28.12 (22.64 to 35.16) | 26.97 (21.78 to 33.61) | -0.74 (-1.02 to -0.45) |
| Kenya | 0.85 (0.71 to 1.02) | 1.16 (0.99 to 1.39) | 0.67 (0.53 to 0.82) | 0.36 (0.22 to 0.53) | 0.84 (0.5 to 1.26) | 0.31 (0.22 to 0.40) | 12.85 (8.71 to 18.65) | 21.31 (13.76 to 30.78) | 0.26 (0.16 to 0.36) |
| Kiribati | 0.51 (0.42 to 0.62) | 0.58 (0.48 to 0.7) | 0.56 (0.48 to 0.64) | 0.62 (0.35 to 1.08) | 1.08 (0.68 to 1.67) | -2.13 (-2.29 to -1.98) | 24.95 (11.93 to 46.26) | 31.83 (17.58 to 55.4) | -2.07 (-2.20 to -1.93) |
| Kuwait | 4.41 (3.63 to 5.41) | 3.72 (3.09 to 4.54) | 0.41 (0.30 to 0.51) | 0.09 (0.07 to 0.12) | 0.15 (0.12 to 0.2) | -0.66 (-1.06 to -0.25) | 12.61 (8.83 to 17.05) | 11.73 (8.74 to 15.53) | -0.33 (-0.50 to -0.15) |
| Kyrgyzstan | 5.97 (5.04 to 7.04) | 6.39 (5.41 to 7.54) | 0.12 (0.06 to 0.18) | 0.15 (0.12 to 0.19) | 0.19 (0.16 to 0.25) | -3.52 (-3.86 to -3.19) | 15.78 (12.07 to 20.28) | 17.37 (13.34 to 22.37) | -2.11 (-2.28 to -1.94) |
| Lao People's Democratic Republic | 0.49 (0.4 to 0.6) | 0.51 (0.42 to 0.63) | 1.04 (0.98 to 1.10) | 0.15 (0.08 to 0.24) | 0.26 (0.14 to 0.41) | -2.40 (-2.60 to -2.19) | 5.41 (3.26 to 8.53) | 6.97 (4.13 to 11.24) | -2.47 (-2.65 to -2.30) |
| Latvia | 8.06 (7.03 to 9.34) | 7.15 (6.25 to 8.09) | 0.53 (0.28 to 0.78) | 0.71 (0.57 to 0.91) | 0.37 (0.3 to 0.48) | 0.22 (-0.08 to 0.51) | 40.66 (31.58 to 51.22) | 28.66 (21.84 to 36.61) | 0.14 (-0.02 to 0.29) |
| Lebanon | 3.74 (3.1 to 4.59) | 3.56 (2.97 to 4.37) | 1.39 (1.29 to 1.49) | 0.15 (0.08 to 0.23) | 0.15 (0.08 to 0.23) | 0.32 (0.10 to 0.55) | 10.54 (7.6 to 14.24) | 10.18 (7.34 to 13.75) | 0.79 (0.65 to 0.93) |
| Lesotho | 1.17 (0.98 to 1.42) | 1.36 (1.14 to 1.65) | 0.47 (0.43 to 0.51) | 0.41 (0.28 to 0.59) | 0.69 (0.48 to 0.98) | 1.08 (0.85 to 1.31) | 15.58 (10.04 to 22.95) | 20.29 (13.61 to 29.06) | 1.06 (0.87 to 1.26) |
| Liberia | 0.89 (0.74 to 1.08) | 1.18 (1 to 1.42) | 0.43 (0.19 to 0.66) | 0.22 (0.12 to 0.35) | 0.42 (0.26 to 0.62) | -0.84 (-1.09 to -0.60) | 10.23 (5.65 to 17.39) | 13.96 (8.48 to 21.59) | -1.16 (-1.44 to -0.88) |
| Libya | 2.64 (2.17 to 3.26) | 2.41 (2.01 to 2.96) | 1.02 (0.72 to 1.32) | 0.16 (0.11 to 0.22) | 0.22 (0.15 to 0.31) | 0.11 (-0.02 to 0.24) | 8.83 (6.48 to 11.61) | 9.05 (6.69 to 11.82) | 0.21 (0.11 to 0.31) |
| Lithuania | 9.22 (8.77 to 9.69) | 8.26 (7.85 to 8.65) | 1.22 (1.15 to 1.29) | 0.64 (0.49 to 0.81) | 0.33 (0.26 to 0.42) | 0.13 (-0.20 to 0.47) | 39.53 (30.81 to 49.22) | 28.75 (21.98 to 36.27) | 0.49 (0.33 to 0.65) |
| Luxembourg | 17.98 (15.84 to 20.65) | 15.55 (13.6 to 17.92) | -0.49 (-0.65 to -0.33) | 1.77 (1.3 to 2.11) | 0.97 (0.72 to 1.16) | 0.55 (0.30 to 0.80) | 60.73 (47.94 to 76.19) | 43.9 (33.95 to 55.68) | 0.27 (0.15 to 0.38) |
| Madagascar | 0.78 (0.65 to 0.95) | 1.06 (0.9 to 1.29) | 0.30 (0.27 to 0.32) | 0.26 (0.18 to 0.39) | 0.59 (0.4 to 0.88) | 0.45 (0.34 to 0.56) | 11.24 (7.62 to 16.39) | 16.98 (11.97 to 24.64) | -0.03 (-0.18 to 0.13) |
| Malawi | 0.75 (0.62 to 0.92) | 1.1 (0.92 to 1.34) | 0.53 (0.47 to 0.60) | 0.3 (0.21 to 0.42) | 0.67 (0.46 to 0.97) | -0.26 (-0.39 to -0.12) | 12.6 (8.35 to 19.22) | 19.08 (13.47 to 26.73) | -0.85 (-1.02 to -0.69) |
| Malaysia | 0.8 (0.71 to 0.91) | 0.76 (0.67 to 0.85) | 1.60 (1.47 to 1.73) | 0.13 (0.1 to 0.18) | 0.17 (0.12 to 0.23) | -0.75 (-1.06 to -0.43) | 4.58 (3.48 to 5.8) | 4.92 (3.71 to 6.26) | -0.54 (-0.73 to -0.35) |
| Maldives | 0.57 (0.46 to 0.69) | 0.51 (0.43 to 0.61) | 1.26 (1.17 to 1.34) | 0.18 (0.13 to 0.23) | 0.3 (0.23 to 0.4) | -2.37 (-2.52 to -2.22) | 5.87 (4.58 to 7.69) | 7.2 (5.69 to 9.23) | -2.50 (-2.68 to -2.32) |
| Mali | 0.65 (0.55 to 0.79) | 1.02 (0.87 to 1.24) | 0.24 (0.11 to 0.37) | 0.25 (0.12 to 0.42) | 0.47 (0.26 to 0.71) | -0.28 (-0.46 to -0.10) | 12.54 (5.05 to 23.44) | 16.53 (8.45 to 26.83) | -0.64 (-0.80 to -0.48) |
| Malta | 9.98 (8.75 to 11.54) | 8.92 (7.75 to 10.47) | 0.32 (0.24 to 0.39) | 1.36 (1.05 to 1.64) | 0.62 (0.48 to 0.74) | 0.78 (0.57 to 0.99) | 33.83 (27.94 to 40.47) | 22.28 (18.12 to 27.35) | 0.40 (0.30 to 0.50) |
| Marshall Islands | 0.58 (0.48 to 0.71) | 0.63 (0.53 to 0.76) | 1.14 (1.02 to 1.25) | 0.5 (0.29 to 0.83) | 0.83 (0.5 to 1.31) | -1.90 (-2.04 to -1.76) | 20.73 (11.23 to 35.95) | 24.94 (14.63 to 40.72) | -1.80 (-1.93 to -1.68) |
| Mauritania | 0.84 (0.7 to 1.02) | 1.12 (0.94 to 1.36) | 0.06 (-0.12 to 0.24) | 0.23 (0.15 to 0.33) | 0.41 (0.28 to 0.57) | -2.01 (-2.18 to -1.84) | 9.38 (6.1 to 13.52) | 13.42 (9.08 to 18.7) | -1.97 (-2.14 to -1.80) |
| Mauritius | 0.69 (0.58 to 0.82) | 0.59 (0.5 to 0.71) | 0.81 (0.78 to 0.84) | 0.27 (0.2 to 0.34) | 0.22 (0.17 to 0.28) | 1.44 (1.10 to 1.78) | 9.14 (7.04 to 11.49) | 7.7 (5.86 to 9.69) | 1.88 (1.53 to 2.23) |
| Mexico | 3.16 (2.75 to 3.63) | 3.06 (2.67 to 3.52) | -0.91 (-1.09 to -0.72) | 0.42 (0.33 to 0.5) | 0.45 (0.36 to 0.54) | 1.68 (1.33 to 2.02) | 16.91 (13.92 to 20.26) | 17.13 (14.12 to 20.45) | 0.68 (0.41 to 0.96) |
| Micronesia (Federated States of) | 0.63 (0.52 to 0.76) | 0.67 (0.56 to 0.81) | 1.12 (1.03 to 1.21) | 0.52 (0.27 to 0.93) | 0.8 (0.48 to 1.32) | -2.09 (-2.29 to -1.88) | 19.69 (9.63 to 37.34) | 23.88 (12.56 to 43.11) | -2.07 (-2.24 to -1.89) |
| Monaco | 11.2 (9.53 to 13.24) | 9.53 (8.11 to 11.17) | 0.03 (-0.02 to 0.07) | 1.07 (0.78 to 1.39) | 0.36 (0.27 to 0.47) | -0.07 (-0.15 to 0.01) | 37.09 (28.25 to 47.34) | 23.1 (17.08 to 29.93) | 0.08 (0.06 to 0.10) |
| Mongolia | 7.41 (6.2 to 8.86) | 7.35 (6.2 to 8.69) | 0.58 (0.55 to 0.61) | 0.35 (0.25 to 0.48) | 0.45 (0.33 to 0.61) | -2.10 (-2.26 to -1.94) | 24.03 (18.31 to 30.35) | 24.75 (19.13 to 31.11) | -1.88 (-2.00 to -1.75) |
| Montenegro | 10.75 (9.18 to 12.7) | 9.46 (8.02 to 11.15) | 0.34 (0.24 to 0.43) | 0.42 (0.33 to 0.53) | 0.3 (0.23 to 0.37) | 0.27 (0.08 to 0.46) | 35.66 (26.57 to 46.45) | 28.38 (20.94 to 37.02) | -0.08 (-0.14 to -0.02) |
| Morocco | 1.67 (1.41 to 1.98) | 1.61 (1.36 to 1.91) | 0.32 (0.26 to 0.39) | 0.16 (0.12 to 0.22) | 0.21 (0.15 to 0.28) | -0.17 (-0.38 to 0.04) | 7.27 (5.53 to 9.38) | 7.6 (5.86 to 9.68) | -0.09 (-0.21 to 0.02) |
| Mozambique | 0.65 (0.54 to 0.78) | 1 (0.85 to 1.19) | 0.74 (0.70 to 0.77) | 0.36 (0.24 to 0.52) | 0.84 (0.58 to 1.2) | 0.94 (0.77 to 1.12) | 15.25 (9.84 to 24.37) | 24.35 (16.57 to 34.27) | 0.62 (0.41 to 0.84) |
| Myanmar | 0.56 (0.46 to 0.69) | 0.55 (0.46 to 0.68) | 1.22 (1.15 to 1.30) | 0.11 (0.08 to 0.15) | 0.13 (0.1 to 0.17) | -1.95 (-2.07 to -1.83) | 4.13 (3.07 to 5.88) | 4.34 (3.28 to 6.04) | -2.29 (-2.41 to -2.17) |
| Namibia | 1.14 (0.95 to 1.39) | 1.37 (1.16 to 1.68) | 0.32 (0.24 to 0.41) | 0.37 (0.26 to 0.54) | 0.63 (0.45 to 0.87) | -0.61 (-0.80 to -0.41) | 13.87 (9.3 to 20.74) | 18.09 (12.71 to 26.34) | -0.52 (-0.63 to -0.41) |
| Nauru | 0.54 (0.44 to 0.66) | 0.64 (0.54 to 0.77) | 1.18 (1.03 to 1.33) | 0.42 (0.19 to 0.78) | 0.92 (0.54 to 1.45) | -1.85 (-2.09 to -1.61) | 20.07 (8.62 to 38.82) | 27.18 (13.97 to 47.82) | -1.87 (-2.08 to -1.65) |
| Nepal | 2.05 (1.69 to 2.51) | 2.24 (1.85 to 2.76) | 1.03 (0.96 to 1.09) | 0.38 (0.23 to 0.63) | 0.59 (0.36 to 0.98) | -1.88 (-2.06 to -1.69) | 12.56 (8.82 to 19.01) | 15.65 (10.93 to 23.88) | -2.15 (-2.36 to -1.94) |
| Netherlands | 8.51 (7.4 to 9.86) | 7.21 (6.24 to 8.42) | -1.79 (-2.43 to -1.15) | 4.56 (3.39 to 5.28) | 2.08 (1.58 to 2.4) | 1.35 (0.87 to 1.84) | 70.53 (59.72 to 80.03) | 41.11 (35.2 to 47.09) | -0.50 (-0.93 to -0.06) |
| New Zealand | 24.3 (21.67 to 27.35) | 22.45 (19.83 to 25.48) | 0.26 (0.21 to 0.31) | 0.7 (0.58 to 0.87) | 0.38 (0.32 to 0.46) | -1.70 (-2.05 to -1.34) | 39.11 (29.52 to 50.14) | 31.98 (23.24 to 41.73) | -0.32 (-0.42 to -0.22) |
| Nicaragua | 1.91 (1.62 to 2.26) | 2.06 (1.77 to 2.43) | -0.44 (-0.52 to -0.36) | 0.11 (0.09 to 0.14) | 0.17 (0.13 to 0.21) | 0.48 (0.30 to 0.67) | 6.82 (5.22 to 8.53) | 8.26 (6.41 to 10.34) | 0.12 (-0.02 to 0.25) |
| Niger | 0.53 (0.43 to 0.64) | 0.9 (0.76 to 1.09) | 0.02 (-0.10 to 0.14) | 0.24 (0.11 to 0.41) | 0.5 (0.29 to 0.79) | -0.71 (-0.92 to -0.51) | 12.17 (4.81 to 23.7) | 17.16 (9.15 to 26.85) | -1.08 (-1.30 to -0.86) |
| Nigeria | 0.8 (0.67 to 0.96) | 1.2 (1.02 to 1.45) | 0.44 (0.27 to 0.61) | 0.26 (0.14 to 0.42) | 0.52 (0.28 to 0.83) | -0.11 (-0.21 to -0.02) | 12.47 (6.99 to 19.77) | 17.08 (10.24 to 26.72) | -0.43 (-0.51 to -0.35) |
| Niue | 0.8 (0.67 to 0.96) | 0.73 (0.61 to 0.87) | 1.36 (1.24 to 1.48) | 0.76 (0.46 to 1.07) | 0.63 (0.38 to 0.91) | -1.75 (-1.86 to -1.63) | 20.88 (11.86 to 31.13) | 19 (10.29 to 29.66) | -1.92 (-2.07 to -1.78) |
| North Macedonia | 11.77 (10.03 to 13.81) | 9.96 (8.49 to 11.66) | 0.32 (0.26 to 0.38) | 0.23 (0.16 to 0.29) | 0.18 (0.13 to 0.23) | -0.21 (-0.36 to -0.07) | 29.5 (21.31 to 39.22) | 23.32 (16.99 to 31.08) | 0.00 (-0.04 to 0.05) |
| Northern Mariana Islands | 0.88 (0.75 to 1.06) | 0.74 (0.63 to 0.89) | 0.79 (0.69 to 0.90) | 0.36 (0.24 to 0.68) | 0.38 (0.26 to 0.73) | -4.76 (-5.32 to -4.19) | 12.06 (8.4 to 22.25) | 11.56 (7.82 to 20.63) | -4.28 (-4.78 to -3.78) |
| Norway | 39.51 (34.29 to 45.47) | 36.64 (31.66 to 42.06) | 0.79 (0.67 to 0.91) | 0.71 (0.59 to 1.03) | 0.38 (0.32 to 0.52) | -0.86 (-1.19 to -0.53) | 101.21 (71.69 to 135.43) | 80.37 (56.25 to 108.56) | 0.17 (0.01 to 0.33) |
| Oman | 3.12 (2.51 to 3.78) | 2.73 (2.27 to 3.27) | 0.06 (-0.11 to 0.22) | 0.08 (0.06 to 0.11) | 0.23 (0.15 to 0.34) | 0.58 (0.34 to 0.82) | 8.19 (5.91 to 10.82) | 9.96 (7.66 to 12.72) | 0.17 (0.00 to 0.35) |
| Pakistan | 1.74 (1.42 to 2.16) | 2.3 (1.91 to 2.86) | 0.69 (0.65 to 0.74) | 0.29 (0.2 to 0.44) | 0.62 (0.41 to 0.99) | -0.87 (-0.93 to -0.80) | 12.18 (8.36 to 17.13) | 17.8 (12.91 to 25.37) | -0.71 (-0.77 to -0.66) |
| Palau | 0.92 (0.77 to 1.11) | 0.76 (0.64 to 0.91) | 1.11 (1.02 to 1.20) | 0.6 (0.26 to 0.97) | 0.62 (0.29 to 0.97) | -1.36 (-1.47 to -1.24) | 21.36 (9.03 to 35.97) | 19.34 (8.32 to 32.27) | -1.21 (-1.31 to -1.10) |
| Palestine | 2.46 (2.04 to 2.92) | 2.94 (2.47 to 3.5) | 1.11 (0.92 to 1.30) | 0.08 (0.06 to 0.13) | 0.2 (0.15 to 0.29) | -2.83 (-3.48 to -2.18) | 6.47 (4.75 to 8.93) | 9.47 (7.18 to 12.75) | -1.16 (-1.42 to -0.90) |
| Panama | 2.22 (1.9 to 2.59) | 2.2 (1.88 to 2.56) | -0.63 (-0.82 to -0.43) | 0.6 (0.45 to 0.77) | 0.59 (0.45 to 0.76) | 2.20 (1.67 to 2.73) | 24.09 (18.33 to 31.17) | 24.28 (18.4 to 31.52) | 1.57 (1.10 to 2.05) |
| Papua New Guinea | 0.44 (0.36 to 0.54) | 0.52 (0.44 to 0.64) | 0.93 (0.81 to 1.05) | 0.26 (0.17 to 0.39) | 0.42 (0.27 to 0.63) | -1.02 (-1.10 to -0.93) | 12.75 (7.68 to 20.61) | 14.68 (9.5 to 21.87) | -1.09 (-1.18 to -1.00) |
| Paraguay | 3.21 (2.74 to 3.76) | 3.38 (2.9 to 3.94) | -0.60 (-1.02 to -0.17) | 0.17 (0.12 to 0.23) | 0.21 (0.15 to 0.28) | 1.35 (1.08 to 1.63) | 9.34 (6.94 to 11.99) | 10.3 (7.7 to 13.16) | 0.12 (-0.06 to 0.29) |
| Peru | 1.79 (1.51 to 2.13) | 1.78 (1.5 to 2.11) | 0.00 (-0.22 to 0.22) | 0.22 (0.16 to 0.31) | 0.23 (0.16 to 0.32) | -4.20 (-4.68 to -3.72) | 9.17 (6.79 to 12.34) | 9.31 (6.89 to 12.58) | -5.08 (-5.69 to -4.47) |
| Philippines | 0.59 (0.48 to 0.71) | 0.63 (0.52 to 0.76) | 0.78 (0.68 to 0.89) | 0.1 (0.08 to 0.14) | 0.16 (0.13 to 0.2) | -3.25 (-3.54 to -2.95) | 4.06 (3.39 to 5.33) | 4.87 (4.06 to 6.26) | -2.61 (-2.86 to -2.35) |
| Poland | 15.1 (13.38 to 17.1) | 13.28 (11.82 to 14.95) | -3.85 (-4.56 to -3.13) | 0.94 (0.73 to 1.11) | 0.53 (0.42 to 0.62) | 0.82 (0.68 to 0.96) | 52.25 (40.59 to 65.21) | 38.44 (29.32 to 49.04) | -2.70 (-3.28 to -2.11) |
| Portugal | 10.44 (9.62 to 11.34) | 9.37 (8.55 to 10.26) | 1.51 (1.22 to 1.80) | 2.87 (1.49 to 3.41) | 1.06 (0.57 to 1.24) | 2.38 (1.99 to 2.77) | 50.06 (32.93 to 58.73) | 27.82 (20.23 to 33.05) | 1.24 (1.07 to 1.41) |
| Puerto Rico | 3.47 (3.07 to 3.9) | 2.93 (2.62 to 3.25) | 0.48 (0.33 to 0.63) | 0.51 (0.36 to 0.8) | 0.27 (0.19 to 0.41) | -3.70 (-4.28 to -3.10) | 16.81 (12.94 to 22.75) | 12.28 (9.43 to 16.08) | -2.69 (-3.09 to -2.29) |
| Qatar | 4.37 (3.56 to 5.31) | 3.55 (3 to 4.28) | 0.90 (0.83 to 0.96) | 0.07 (0.04 to 0.11) | 0.34 (0.21 to 0.51) | 0.52 (0.07 to 0.98) | 10.33 (7.17 to 14.37) | 13.06 (9.28 to 17.59) | 0.13 (-0.12 to 0.37) |
| Republic of Korea | 8.25 (7.9 to 8.59) | 7.33 (7.03 to 7.63) | 1.94 (1.57 to 2.30) | 0.44 (0.34 to 0.59) | 0.3 (0.23 to 0.39) | -7.96 (-8.67 to -7.25) | 19.57 (14.66 to 24.99) | 15 (11.13 to 19.33) | -3.63 (-4.02 to -3.23) |
| Republic of Moldova | 5.25 (4.86 to 5.69) | 4.51 (4.16 to 4.86) | 0.38 (0.18 to 0.58) | 0.48 (0.39 to 0.57) | 0.33 (0.27 to 0.39) | -0.60 (-0.96 to -0.25) | 27.09 (22.22 to 32.78) | 20.72 (16.98 to 25.12) | -0.20 (-0.52 to 0.13) |
| Romania | 6.17 (5.64 to 6.79) | 5.63 (5.11 to 6.27) | 0.59 (0.34 to 0.83) | 0.38 (0.31 to 0.48) | 0.23 (0.18 to 0.28) | -0.65 (-1.01 to -0.29) | 22.68 (17.7 to 28.38) | 17.97 (13.92 to 22.6) | -0.63 (-0.89 to -0.37) |
| Russian Federation | 9.89 (8.5 to 11.69) | 7.86 (6.75 to 9.25) | -0.06 (-0.16 to 0.05) | 0.86 (0.7 to 1) | 0.56 (0.46 to 0.66) | -0.40 (-0.74 to -0.06) | 38.53 (31.44 to 47.25) | 28.04 (22.97 to 34.48) | -0.52 (-0.70 to -0.35) |
| Rwanda | 0.8 (0.66 to 0.97) | 1.04 (0.87 to 1.25) | 0.40 (0.30 to 0.50) | 0.33 (0.21 to 0.49) | 0.65 (0.42 to 0.92) | -1.79 (-2.06 to -1.51) | 14.48 (9.09 to 23.74) | 19.33 (12.66 to 28.48) | -1.92 (-2.15 to -1.69) |
| Saint Kitts and Nevis | 3.43 (2.89 to 4.16) | 2.93 (2.48 to 3.54) | 0.69 (0.47 to 0.90) | 1.48 (1.06 to 2.15) | 1.44 (1.06 to 2.05) | -2.01 (-2.26 to -1.76) | 47.78 (32.3 to 69.09) | 42.16 (29.05 to 61) | -2.07 (-2.32 to -1.82) |
| Saint Lucia | 3.09 (2.6 to 3.75) | 2.64 (2.22 to 3.19) | 0.28 (0.11 to 0.45) | 0.75 (0.59 to 0.98) | 0.65 (0.51 to 0.84) | -2.24 (-2.69 to -1.78) | 25.73 (20.63 to 32.33) | 22.26 (17.86 to 28.14) | -1.84 (-2.19 to -1.49) |
| Saint Vincent and the Grenadines | 3.12 (2.61 to 3.76) | 2.8 (2.35 to 3.35) | 0.49 (0.27 to 0.70) | 1.37 (1.09 to 1.73) | 1.22 (0.98 to 1.53) | -0.56 (-0.70 to -0.41) | 43.38 (35.24 to 54.37) | 38.61 (31.46 to 48.07) | -0.80 (-0.94 to -0.67) |
| Samoa | 0.53 (0.44 to 0.64) | 0.6 (0.51 to 0.73) | 1.10 (0.77 to 1.44) | 0.43 (0.31 to 0.6) | 0.63 (0.45 to 0.85) | -1.64 (-1.80 to -1.47) | 14.35 (9.16 to 21.31) | 17.97 (11.92 to 26.18) | -1.62 (-1.75 to -1.49) |
| San Marino | 10.42 (8.95 to 12.1) | 9.06 (7.71 to 10.59) | 0.20 (0.15 to 0.25) | 0.91 (0.55 to 1.35) | 0.36 (0.22 to 0.53) | 0.22 (0.06 to 0.38) | 31.53 (23.27 to 41.65) | 21.79 (15.52 to 28.71) | 0.15 (0.11 to 0.19) |
| Sao Tome and Principe | 0.93 (0.77 to 1.14) | 1.15 (0.96 to 1.41) | 0.72 (0.50 to 0.94) | 0.17 (0.12 to 0.28) | 0.31 (0.21 to 0.5) | -1.58 (-1.93 to -1.24) | 7.57 (5.33 to 11.59) | 10.34 (7.41 to 15.57) | -1.71 (-2.03 to -1.38) |
| Saudi Arabia | 3.14 (2.6 to 3.79) | 2.66 (2.24 to 3.18) | -0.14 (-0.29 to 0.01) | 0.08 (0.05 to 0.11) | 0.15 (0.11 to 0.21) | -0.60 (-0.71 to -0.48) | 8.9 (6.39 to 12.25) | 8.9 (6.65 to 11.75) | -0.18 (-0.25 to -0.10) |
| Senegal | 0.87 (0.72 to 1.06) | 1.18 (1 to 1.47) | 0.34 (0.21 to 0.47) | 0.24 (0.14 to 0.38) | 0.43 (0.27 to 0.65) | -1.00 (-1.21 to -0.78) | 10.09 (6.02 to 15.71) | 14.15 (8.71 to 21.53) | -1.12 (-1.32 to -0.91) |
| Serbia | 14.22 (12.22 to 16.55) | 12.68 (10.96 to 14.68) | 0.78 (0.55 to 1.01) | 1.29 (0.85 to 1.68) | 0.76 (0.5 to 0.97) | 2.49 (2.05 to 2.94) | 54.2 (41.1 to 68.56) | 39.47 (29.72 to 49.82) | 0.83 (0.67 to 0.98) |
| Seychelles | 0.68 (0.57 to 0.82) | 0.61 (0.51 to 0.73) | 0.67 (0.62 to 0.73) | 0.22 (0.16 to 0.29) | 0.22 (0.16 to 0.3) | -1.44 (-1.65 to -1.23) | 6.73 (5.16 to 8.43) | 6.28 (4.77 to 7.85) | -1.35 (-1.51 to -1.19) |
| Sierra Leone | 0.81 (0.67 to 0.98) | 1.12 (0.95 to 1.37) | 0.15 (-0.01 to 0.32) | 0.24 (0.12 to 0.38) | 0.43 (0.24 to 0.61) | 0.20 (-0.07 to 0.48) | 11.84 (5.52 to 19.99) | 15.3 (8.12 to 23.35) | 0.00 (-0.27 to 0.26) |
| Singapore | 3.79 (3.14 to 4.65) | 3.21 (2.68 to 3.9) | -0.35 (-0.57 to -0.13) | 0.09 (0.07 to 0.14) | 0.07 (0.06 to 0.11) | -3.18 (-3.41 to -2.95) | 10.81 (7.54 to 14.79) | 8.16 (5.69 to 11.25) | -1.52 (-1.73 to -1.31) |
| Slovakia | 10.47 (9.37 to 11.87) | 9.7 (8.69 to 10.81) | -0.17 (-0.27 to -0.08) | 1.07 (0.74 to 1.4) | 0.68 (0.48 to 0.9) | 1.56 (1.21 to 1.91) | 52.52 (39.99 to 65.41) | 40.31 (30.39 to 50.67) | 0.34 (0.19 to 0.49) |
| Slovenia | 13.35 (11.8 to 15.24) | 12.39 (10.96 to 14.1) | 0.45 (0.37 to 0.54) | 0.89 (0.63 to 1.19) | 0.39 (0.28 to 0.52) | -1.39 (-2.13 to -0.65) | 47.12 (34.99 to 60.42) | 34.27 (24.57 to 44.81) | -0.42 (-0.67 to -0.17) |
| Solomon Islands | 0.45 (0.36 to 0.56) | 0.56 (0.46 to 0.68) | 0.88 (0.79 to 0.97) | 0.3 (0.17 to 0.51) | 0.53 (0.33 to 0.79) | -1.42 (-1.56 to -1.29) | 13.09 (7.28 to 23.83) | 17.28 (10.22 to 29.48) | -1.38 (-1.51 to -1.25) |
| Somalia | 0.49 (0.4 to 0.61) | 0.76 (0.63 to 0.94) | 0.02 (-0.01 to 0.05) | 0.2 (0.1 to 0.35) | 0.48 (0.3 to 0.79) | -0.45 (-0.53 to -0.37) | 9.76 (4.56 to 19.6) | 14.47 (8.44 to 24.4) | -0.63 (-0.69 to -0.56) |
| South Africa | 1.34 (1.14 to 1.6) | 1.37 (1.17 to 1.64) | 0.13 (0.06 to 0.20) | 0.3 (0.22 to 0.35) | 0.39 (0.3 to 0.47) | 0.13 (-0.38 to 0.64) | 11.05 (8.93 to 12.74) | 12.17 (9.76 to 14.04) | -0.56 (-0.99 to -0.14) |
| South Sudan | 0.68 (0.57 to 0.82) | 0.99 (0.84 to 1.2) | 0.25 (0.22 to 0.28) | 0.31 (0.17 to 0.66) | 0.65 (0.36 to 1.4) | -0.05 (-0.09 to 0.00) | 14.17 (6.86 to 28.28) | 19.12 (11.06 to 39.88) | -0.45 (-0.58 to -0.32) |
| Spain | 14.77 (14.25 to 15.34) | 14.01 (13.46 to 14.57) | 0.86 (0.76 to 0.97) | 0.82 (0.66 to 1.3) | 0.35 (0.29 to 0.5) | -2.16 (-2.54 to -1.78) | 37.95 (29.41 to 48.03) | 27.31 (20.61 to 34.99) | -0.26 (-0.47 to -0.05) |
| Sri Lanka | 1.23 (1.09 to 1.38) | 1.15 (1.02 to 1.3) | 1.86 (1.62 to 2.11) | 0.1 (0.07 to 0.14) | 0.1 (0.07 to 0.13) | -2.15 (-2.31 to -1.99) | 4.1 (3.13 to 5.25) | 3.77 (2.86 to 4.79) | -1.45 (-1.57 to -1.33) |
| Sudan | 2.29 (1.9 to 2.73) | 2.77 (2.32 to 3.33) | 0.67 (0.63 to 0.72) | 0.12 (0.07 to 0.19) | 0.25 (0.14 to 0.43) | 0.95 (0.78 to 1.11) | 7.36 (5.15 to 10.12) | 10.84 (7.56 to 15.32) | 0.71 (0.58 to 0.84) |
| Suriname | 2.94 (2.45 to 3.55) | 2.76 (2.31 to 3.34) | 0.51 (0.37 to 0.65) | 0.66 (0.5 to 0.86) | 0.66 (0.5 to 0.86) | -1.99 (-2.24 to -1.73) | 23.9 (18.84 to 30.32) | 23 (18.2 to 29.1) | -1.99 (-2.24 to -1.75) |
| Sweden | 31.04 (27.53 to 35.6) | 27.26 (23.97 to 31.28) | 0.66 (0.62 to 0.71) | 0.92 (0.75 to 1.36) | 0.38 (0.31 to 0.56) | -3.16 (-4.26 to -2.06) | 69.62 (50.36 to 93.4) | 53 (37.35 to 71.73) | -0.35 (-0.54 to -0.16) |
| Switzerland | 12.63 (11.32 to 14.42) | 11.45 (10.2 to 12.96) | -0.01 (-0.14 to 0.12) | 1.4 (1.11 to 1.83) | 0.58 (0.47 to 0.75) | -1.03 (-1.80 to -0.26) | 43.38 (33.95 to 53.99) | 29.51 (22.3 to 37.67) | -0.56 (-0.82 to -0.31) |
| Syrian Arab Republic | 3.1 (2.59 to 3.75) | 3.16 (2.63 to 3.83) | 0.25 (0.05 to 0.46) | 0.25 (0.14 to 0.36) | 0.36 (0.19 to 0.52) | -1.00 (-1.20 to -0.80) | 11.41 (8.25 to 15.15) | 12.75 (9 to 16.93) | -0.46 (-0.53 to -0.39) |
| Taiwan (Province of China) | 1.99 (1.78 to 2.23) | 1.72 (1.54 to 1.93) | 3.87 (3.46 to 4.28) | 0.84 (0.65 to 1.12) | 0.5 (0.38 to 0.65) | -2.08 (-3.37 to -0.78) | 18.36 (14.81 to 22.93) | 12.4 (10.06 to 15.44) | -0.85 (-1.78 to 0.10) |
| Tajikistan | 5.46 (4.63 to 6.45) | 6.23 (5.32 to 7.3) | 0.16 (0.09 to 0.23) | 0.19 (0.13 to 0.27) | 0.3 (0.22 to 0.41) | -0.79 (-0.88 to -0.70) | 17.34 (12.85 to 23.02) | 20.6 (15.55 to 26.9) | -0.89 (-1.10 to -0.69) |
| Thailand | 0.53 (0.44 to 0.65) | 0.46 (0.38 to 0.56) | 1.11 (1.03 to 1.18) | 0.16 (0.11 to 0.21) | 0.12 (0.08 to 0.15) | -2.46 (-2.78 to -2.14) | 4.67 (3.5 to 6.08) | 3.64 (2.76 to 4.68) | -2.40 (-2.71 to -2.09) |
| Timor-Leste | 0.44 (0.35 to 0.53) | 0.51 (0.42 to 0.62) | 1.50 (1.36 to 1.65) | 0.21 (0.11 to 0.36) | 0.36 (0.2 to 0.61) | -0.57 (-0.70 to -0.44) | 6.47 (3.59 to 10.34) | 9.14 (5.17 to 14.89) | -0.95 (-1.13 to -0.77) |
| Togo | 0.94 (0.78 to 1.13) | 1.23 (1.04 to 1.48) | 0.58 (0.43 to 0.73) | 0.27 (0.17 to 0.38) | 0.48 (0.32 to 0.68) | -0.05 (-0.31 to 0.21) | 11.87 (7.44 to 16.68) | 16.3 (10.74 to 22.75) | -0.23 (-0.48 to 0.01) |
| Tokelau | 0.61 (0.51 to 0.73) | 0.64 (0.54 to 0.78) | 0.57 (0.34 to 0.80) | 0.51 (0.37 to 0.7) | 0.58 (0.42 to 0.78) | -1.72 (-1.88 to -1.57) | 15.21 (10.49 to 21.64) | 16.11 (11.19 to 22.98) | -1.68 (-1.80 to -1.56) |
| Tonga | 0.58 (0.48 to 0.7) | 0.66 (0.55 to 0.78) | 1.30 (1.13 to 1.48) | 0.61 (0.45 to 0.81) | 0.76 (0.56 to 1.02) | -1.73 (-1.92 to -1.54) | 18.4 (13.11 to 26.06) | 21.58 (15.55 to 30.1) | -1.63 (-1.83 to -1.44) |
| Trinidad and Tobago | 3.77 (3.19 to 4.58) | 3.19 (2.68 to 3.86) | 0.39 (0.18 to 0.60) | 0.37 (0.26 to 0.5) | 0.29 (0.21 to 0.39) | -0.98 (-1.25 to -0.72) | 15.5 (11.7 to 20.08) | 12.54 (9.51 to 16.14) | -0.66 (-0.84 to -0.48) |
| Tunisia | 3.02 (2.49 to 3.68) | 2.73 (2.25 to 3.32) | 0.57 (0.53 to 0.61) | 0.14 (0.09 to 0.2) | 0.14 (0.09 to 0.2) | -0.05 (-0.15 to 0.06) | 10.5 (7.69 to 14.02) | 9.36 (6.89 to 12.4) | 0.27 (0.17 to 0.37) |
| Turkey | 7.33 (6.29 to 8.47) | 6.44 (5.55 to 7.42) | 1.27 (1.06 to 1.48) | 0.4 (0.31 to 0.58) | 0.4 (0.3 to 0.56) | -1.87 (-2.02 to -1.72) | 27.64 (21.21 to 35.52) | 25.05 (19.42 to 32.24) | -0.61 (-0.70 to -0.51) |
| Turkmenistan | 7.53 (6.38 to 8.86) | 7.68 (6.53 to 9.05) | 0.54 (0.44 to 0.64) | 0.28 (0.18 to 0.39) | 0.33 (0.22 to 0.46) | -0.50 (-0.78 to -0.21) | 22.04 (15.83 to 28.29) | 22.9 (16.63 to 29.27) | -0.20 (-0.38 to -0.02) |
| Tuvalu | 0.67 (0.56 to 0.81) | 0.69 (0.57 to 0.83) | 1.23 (1.17 to 1.30) | 0.57 (0.38 to 0.86) | 0.71 (0.47 to 1.05) | -1.60 (-1.73 to -1.47) | 19.25 (11.9 to 30.62) | 21.17 (13.37 to 33.22) | -1.65 (-1.76 to -1.55) |
| Uganda | 0.68 (0.56 to 0.83) | 1.07 (0.9 to 1.3) | 0.65 (0.58 to 0.72) | 0.25 (0.17 to 0.37) | 0.61 (0.4 to 0.92) | -0.03 (-0.17 to 0.10) | 11.19 (7.5 to 17.17) | 17.4 (12.18 to 25.46) | -0.02 (-0.16 to 0.13) |
| Ukraine | 8.23 (7.02 to 9.67) | 6.22 (5.33 to 7.29) | 0.19 (0.10 to 0.28) | 0.59 (0.48 to 0.72) | 0.39 (0.31 to 0.48) | 0.17 (-0.10 to 0.44) | 32.23 (26.04 to 39.08) | 23.62 (18.99 to 28.82) | 0.34 (0.12 to 0.56) |
| United Arab Emirates | 5.63 (4.44 to 7.04) | 4.12 (3.4 to 5.01) | 0.76 (0.59 to 0.94) | 0.14 (0.08 to 0.24) | 0.24 (0.15 to 0.43) | 0.72 (0.25 to 1.20) | 15.16 (10.5 to 21.26) | 12.4 (8.88 to 17.45) | 0.73 (0.52 to 0.94) |
| United Kingdom | 24.83 (22.1 to 28.34) | 23.11 (20.33 to 26.28) | 0.54 (0.47 to 0.61) | 2.67 (2.28 to 2.97) | 1.3 (1.12 to 1.44) | 1.22 (0.84 to 1.59) | 82.07 (67.42 to 98.51) | 59.34 (47.21 to 72.73) | 0.41 (0.25 to 0.57) |
| United Republic of Tanzania | 0.74 (0.61 to 0.9) | 1.05 (0.88 to 1.28) | 0.56 (0.41 to 0.71) | 0.32 (0.2 to 0.46) | 0.6 (0.41 to 0.89) | 0.38 (0.19 to 0.57) | 15.1 (8.79 to 24.83) | 18.91 (12.48 to 26.55) | 0.25 (0.07 to 0.43) |
| United States of America | 26.03 (23.77 to 28.86) | 23.19 (21.18 to 25.62) | -1.00 (-1.44 to -0.55) | 1.8 (1.41 to 1.97) | 1.02 (0.8 to 1.11) | 1.80 (1.52 to 2.07) | 65.64 (53.66 to 78.07) | 49.82 (40.25 to 60.19) | -0.48 (-0.78 to -0.18) |
| United States Virgin Islands | 3.48 (2.92 to 4.2) | 2.91 (2.47 to 3.5) | 0.73 (0.57 to 0.89) | 0.7 (0.53 to 0.9) | 0.45 (0.34 to 0.59) | -2.32 (-2.66 to -1.99) | 22.4 (17.78 to 28.04) | 15.91 (12.54 to 20.17) | -2.03 (-2.27 to -1.78) |
| Uruguay | 1.91 (1.61 to 2.27) | 1.76 (1.47 to 2.09) | 0.85 (0.76 to 0.94) | 0.65 (0.56 to 0.76) | 0.41 (0.35 to 0.47) | 1.10 (0.62 to 1.58) | 16.65 (14.45 to 18.87) | 12.78 (11.11 to 14.58) | 0.63 (0.27 to 0.98) |
| Uzbekistan | 6.57 (5.58 to 7.85) | 6.9 (5.9 to 8.2) | 1.04 (0.95 to 1.13) | 0.22 (0.17 to 0.28) | 0.29 (0.23 to 0.39) | -0.33 (-0.58 to -0.09) | 20.4 (16.01 to 25.24) | 21.9 (17.37 to 27.16) | -0.47 (-0.58 to -0.36) |
| Vanuatu | 0.5 (0.4 to 0.6) | 0.59 (0.49 to 0.71) | 0.99 (0.88 to 1.10) | 0.52 (0.31 to 0.78) | 0.84 (0.52 to 1.24) | -1.74 (-1.94 to -1.54) | 19.44 (11.14 to 31.36) | 25.36 (15.29 to 38.79) | -1.58 (-1.80 to -1.36) |
| Venezuela (Bolivarian Republic of) | 2.33 (1.98 to 2.73) | 2.18 (1.85 to 2.55) | -0.64 (-0.84 to -0.44) | 0.16 (0.11 to 0.21) | 0.15 (0.11 to 0.21) | -2.32 (-2.82 to -1.82) | 9.41 (7.24 to 12.01) | 8.87 (6.84 to 11.26) | -1.36 (-1.66 to -1.05) |
| Viet Nam | 1.54 (1.25 to 1.91) | 1.37 (1.11 to 1.7) | 2.62 (2.11 to 3.13) | 0.18 (0.12 to 0.26) | 0.22 (0.15 to 0.32) | -3.98 (-4.35 to -3.61) | 7.7 (5.72 to 9.96) | 7.6 (5.65 to 9.8) | -2.48 (-2.72 to -2.23) |
| Yemen | 2.05 (1.67 to 2.52) | 2.54 (2.1 to 3.09) | 0.73 (0.69 to 0.77) | 0.14 (0.09 to 0.21) | 0.33 (0.22 to 0.52) | 0.85 (0.73 to 0.97) | 7.69 (5.68 to 10.31) | 12.4 (9.18 to 16.77) | 0.79 (0.66 to 0.93) |
| Zambia | 0.78 (0.64 to 0.95) | 1.11 (0.95 to 1.35) | 0.32 (0.22 to 0.43) | 0.3 (0.21 to 0.42) | 0.7 (0.51 to 0.94) | -0.86 (-1.04 to -0.69) | 13.97 (8.89 to 20.84) | 20.49 (14.47 to 27.66) | -1.05 (-1.14 to -0.96) |
| Zimbabwe | 0.97 (0.8 to 1.18) | 1.3 (1.09 to 1.59) | -0.14 (-0.27 to -0.01) | 0.29 (0.16 to 0.43) | 0.58 (0.35 to 0.86) | 0.96 (0.85 to 1.06) | 12.58 (7.16 to 19.78) | 17.38 (10.28 to 25.35) | 1.22 (0.99 to 1.44) |
